# Supplementary material for: Activity-Dependent Downscaling of Subthreshold Synaptic Inputs during Slow-Wave-Sleep-like Activity In Vivo
Source: Neuron. 2018 Mar 21;97(6):1244–1252.e5. doi: 10.1016/j.neuron.2018.01.047 (PMC5873548; doi:10.1016/j.neuron.2018.01.047)
Supplement: Document S1. Figures S1–S4 [file mmc1.pdf]

Neuron, Volume *97*

Supplemental Information

Activity-Dependent Downscaling of Subthreshold Synaptic Inputs during Slow-Wave-Sleep-like Activity *In Vivo*

Ana González-Rueda, Victor Pedrosa, Rachael C. Feord, Claudia Clopath, and Ole Paulsen

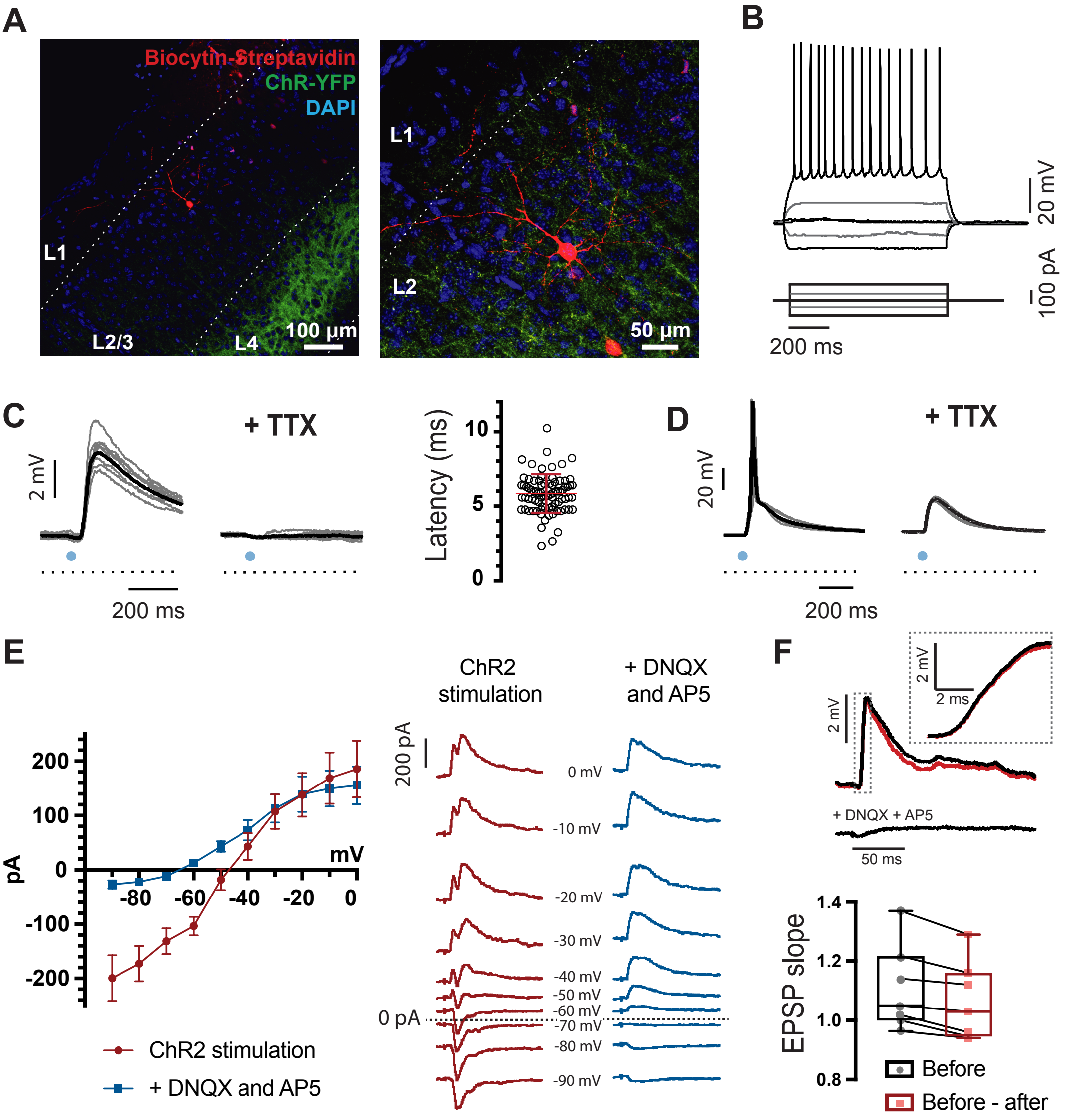

Figure S1 (related to Figure 1): Whole-cell patch clamp of L2/3 neurons in Six3-cre/Ai32 mice.

(**A**) Example L2/3 neuron recorded *in vivo* and filled with biocytin.

(**B**) Example response of the membrane potential of a regular spiking neuron recorded *in vivo* (top) to different steps of current (bottom).

(**C**) Light-evoked EPSPs recorded in L2/3 are completely eliminated when tetrodotoxin (TTX) is added to the superfusate. The average EPSP latency of all recordings is shown (n = 92, mean ± SD).

(**D**) Subthreshold depolarization by direct activation of ChR2 in L4 neurons is unaffected by TTX.

(**E**) Light-evoked EPSC current-voltage curve before (red) and after (blue) DNQX and AP5 application in acute brain slices. Light intensity was previously adjusted to produce a 4 mV EPSP at resting potential in current clamp. The reversal potential of the ChR2-mediated EPSC could be extrapolated at -47 mV and the inhibitory component at -66 mV. n = 7 cells in N = 3 mice.

(**F**) Example light-evoked EPSP before (black top) and after (black bottom)

DNQX and AP5 application in acute brain slices. The subtracted trace (before minus after) is shown in red. n = 7 cells in N = 3 mice. The box-and-whisker plots represent the maximum, upper quartile, mean (cross), median, lower quartile and minimum values and the values for individual neurons are overlaid. The GABA-mediated component of the EPSP could only account for 4% of the measured slope.

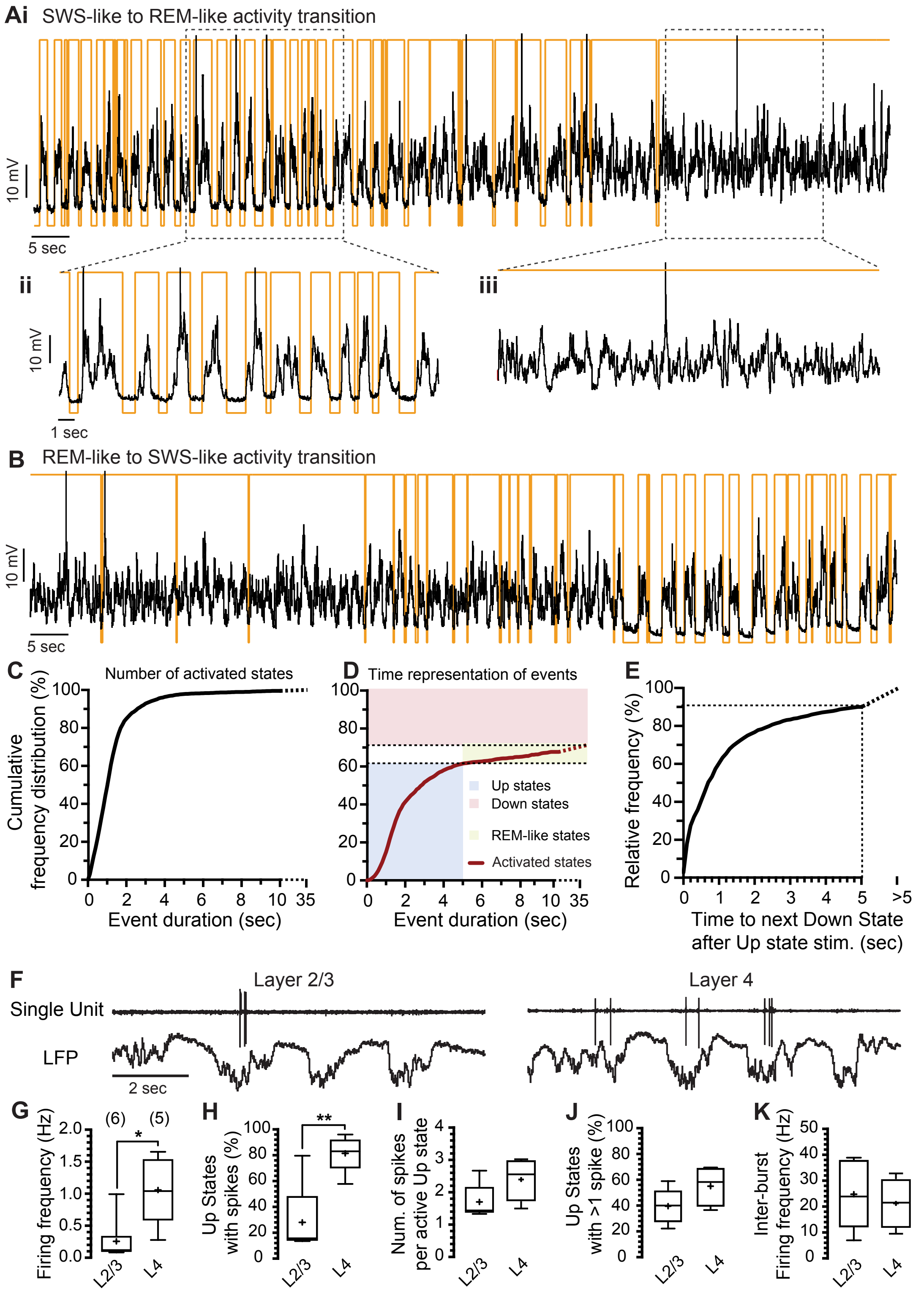

Figure S2 (related to Figure 1): Spontaneous activity in P16-P21 Six3-Cre/Ai32 mice under urethane anesthesia.

(**A**) Example of slow-wave-sleep (SWS)-like to rapid eye movement (REM)-like activity transition (**i**). The Up and Down state transitions are overlaid in orange and a close up of a SWS-like period (**ii**) and REM-like period (**iii**) are shown. Spikes were cut for display.

(**B**) Example of REM-like to SWS-like transition.

(**C**) Cumulative distribution of the number of activated states (Up states and

REM-like states) of different durations. The majority of activated events (84.5%) correspond to Up states lasting <2 seconds and only 2.2% of the events corresponded to putative REM-like states lasting >5 seconds. Data obtained from 30 minute recordings in n = 5 neurons in N = 4 mice.

(**D**) Time representation of Up states, putative REM-like states (lasting >5 seconds) and Down states (as analysed for **C**). Neurons spent 10.1% of the total time in REM-like states.

(**E**) Time to next Down state following Up state stimulation during Up state stimulation protocols (protocols 3, 5, 6 and 7). Only 9% of stimulations were done during putative REM-like states.

(**F**) Example traces of layer (L)2/3 and L4 single unit recordings paired with LFP recordings in L2/3. n = 6 L2/3 and n = 5 L4 neurons recorded in N = 4 mice.

(**G**) Firing frequency (in Hz) of L2/3 and L4 neurons.

(**H**) Percentage of Up states in which L2/3 and L4 were active.

(**I**) Number of spikes per Up state in which L2/3 or L4 were active (respectively).

(**J**) Percentage of Up states in which neurons spiked and produced more than one action potential.

(**K**) Inter-burst frequency when neurons were activated more than once in an Up state.

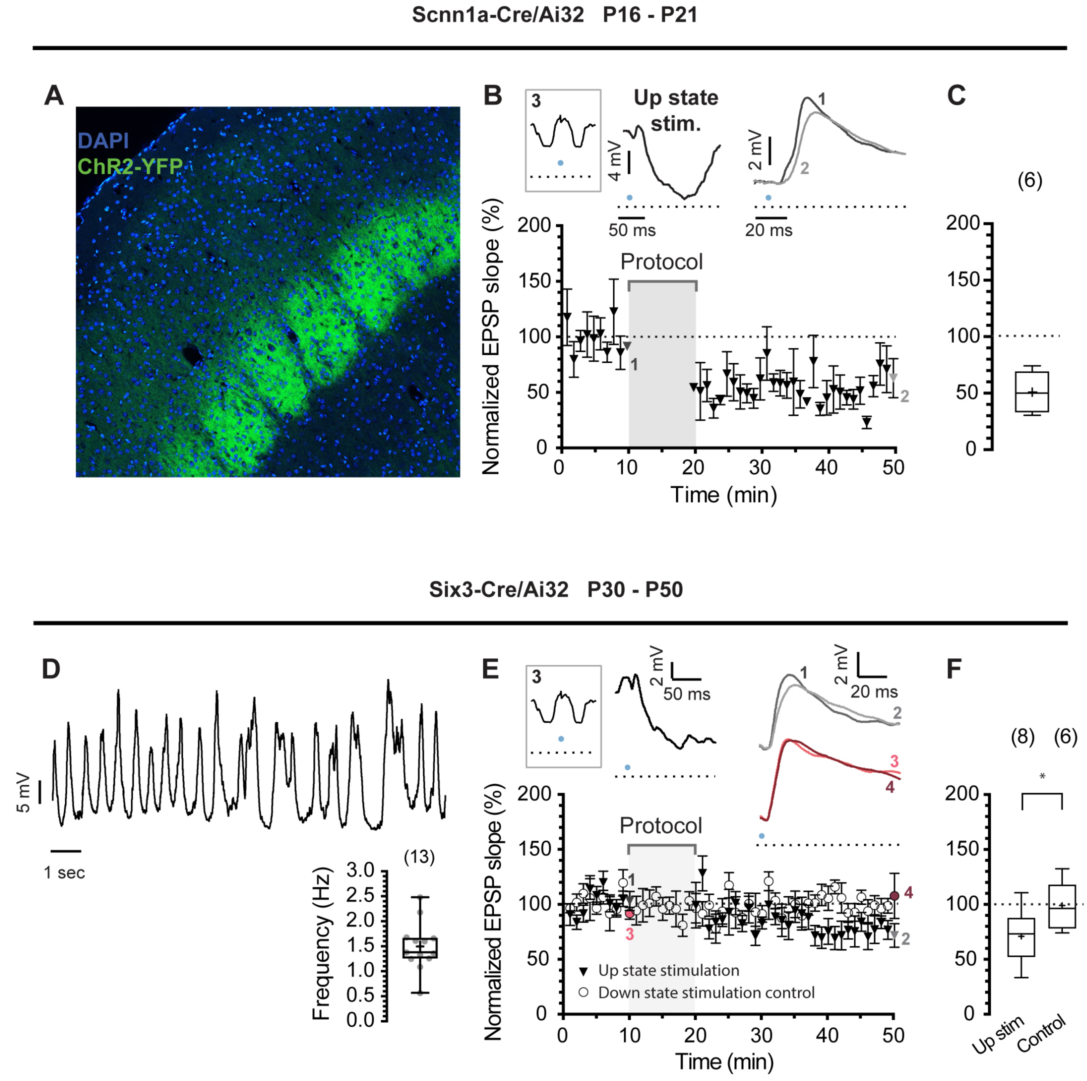

Figure S3 (related to Figure 3): Up state stimulation leads to L4 to L2/3 depression in a different mouse line and at a different age.

(**A**) ChR2 expression pattern in Scnn1a-cre/Ai32 mice.

(**B**) L4 light-stimulation during Up states leads to LTD in Scnn1a-cre/Ai32 mice. Schematic of the stimulation protocol (protocol 3, gray rectangle, top left), representative traces of the plasticity protocol (black trace, top middle) and the average traces from the 10th and 50th minute of an example experiment (1 and 2 respectively) are shown.

(**C**) Summary of result in B. The box-and-whisker plots represent the maximum, upper quartile, mean (cross), median, lower quartile and minimum values (n = 6 cells in N = 6 mice).

(**D**) Example of spontaneous Up and Down state (UDS) activity in P30-P50 Six3-cre/Ai32 mice under urethane anaesthesia. The UDS frequency for all neurons recorded at this age is shown (n = 13 neurons in N = 10 mice).

(**E**) L4 light-stimulation during Up states leads to depression (black triangles, n = 8 neurons in N = 8 mice) while no changes in synaptic weight are observed when presynaptic stimulation is performed during Down states (control, circles, n = 6 neurons in N = 5 mice).

(**F**) Summary of results in E. Two-tailed Student's t test. * $p < 0.05$.

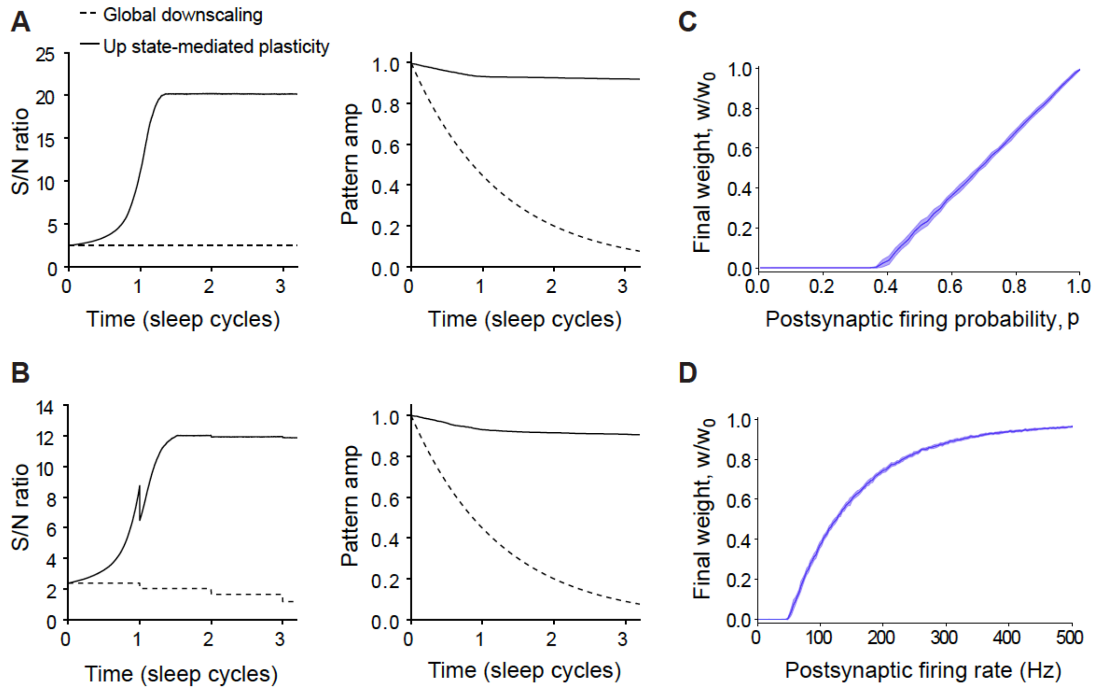

Figure S4 (related to Figure 4): Circuit refinement is independent of specific awake learning rule and synaptic depression can be prevented by postsynaptic activity.

(**A**-**B**) Simulation of a feedforward network composed of 100 presynaptic neurons (representing L4 neurons) projecting onto one single postsynaptic neuron (representing a L2/3 neuron), analogous to simulations in Figure 4. One 'wake' phase of learning was simulated. During this phase, 5 presynaptic neurons received 50% stronger inputs. The synaptic weights at the end of this simulation were used as the initial weight for the sleep phase. These simulations are analogous to the simulations performed in Figure 4F, but with different awake learning rules.

(**A**) Simulated Up state-mediated plasticity preserves and enhances previously stored patterns without learning between sleep cycles. During sleep cycles, all presynaptic neurons received comparable external input and fired at the same rate, on average. Left: evolution of S/N ratio over three sleep cycles. Right: evolution of pattern amplitudes. During sleep cycles, synaptic weights were updated following either the Up state-modulated plasticity (solid lines) or a homogeneous synaptic scaling rule (dashed lines). Between sleep cycles, synaptic weights were kept fixed. Curves show an average over 50 trials.

(**B**) Simulated Up state-mediated plasticity preserves and enhances previously stored patterns when a second pattern is strengthened between sleep cycles. The simulation was performed in the same way as in A but a second pattern was strengthened between sleep cycles. During sleep cycles, all presynaptic neurons received comparable external input and fired at the same rate, on average. Left: evolution of S/N ratio over three sleep cycles. Right: evolution of pattern amplitudes. During sleep cycles, synaptic weights

were updated following either the Up state-modulated plasticity (solid lines) or a homogeneous synaptic scaling rule (dashed lines). Between sleep cycles, the synaptic weights of a second set of presynaptic neurons (pattern 2) were reset to their maximum values. Curves show an average over 50 trials. Our simulations indicate that the Up state-mediated depression leads to circuit refinement independent of the specific rule underlying awake learning.

(**C**-**D**) We simulated a very simple feedforward network, with only one pre- and one postsynaptic neuron. The presynaptic neuron fired at 10 Hz and we assumed that the connection between the neurons was weak enough such that, at this rate, presynaptic spikes would not elicit postsynaptic action potentials.

(**C**) Ratio between final synaptic weight and initial synaptic weight as a function of p, where p is the probability of the postsynaptic neuron to fire within 10 ms after a presynaptic spike (and then prevent synaptic depression). The synapse was updated following the Up state-mediated plasticity. Curves show an average over 50 trials. Shaded areas represent one standard deviation from average.

(**D**) Ratio between final synaptic weight and initial synaptic weight as a function of the postsynaptic firing rate. Both neurons fired independently following a Poisson process. The synapse was updated following the Up state-mediated plasticity. Curves show an average over 50 trials. Shaded areas represent one standard deviation from average.
