# Supplementary material for: Activity-Dependent Downscaling of Subthreshold Synaptic Inputs during Slow-Wave-Sleep-like Activity In Vivo
Source: Neuron. 2018 Mar 21;97(6):1244–1252.e5. doi: 10.1016/j.neuron.2018.01.047 (PMC5873548; doi:10.1016/j.neuron.2018.01.047)
Supplement: Document S2. Article plus Supplemental Information [file mmc2.pdf]

Neuron

Report

Activity-Dependent Downscaling of Subthreshold Synaptic Inputs during Slow-Wave-Sleep-like Activity *In Vivo*

Graphical Abstract

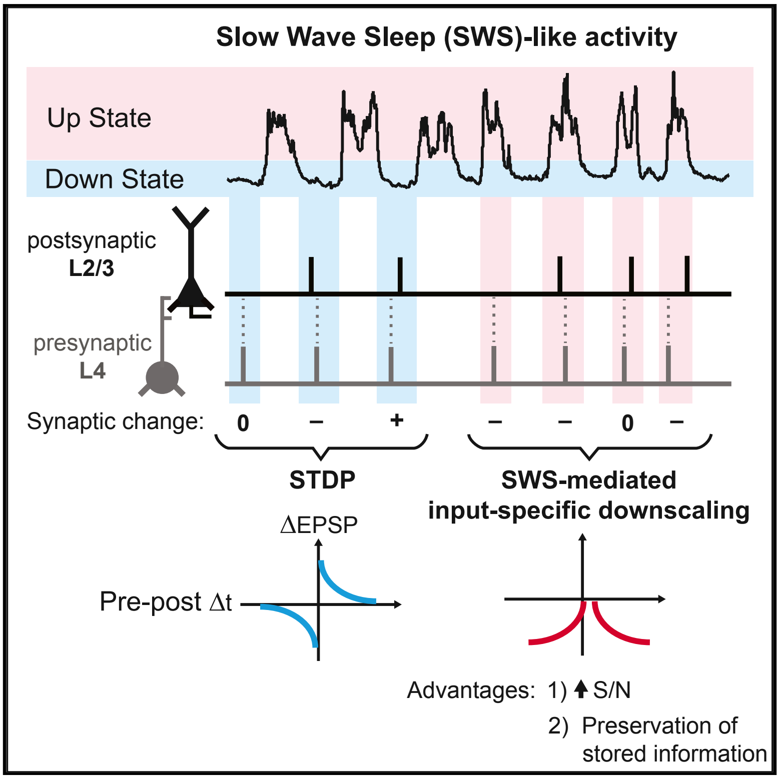

Highlights

- Conventional STDP is seen only during Down states *in vivo*
- During Up states synaptic activation of L4 inputs leads to synaptic depression
- Postsynaptic spikes protect against Up state-mediated synaptic weakening
- These new plasticity rules improve S/N ratio and preserve stored information

Authors

Ana González-Rueda, Victor Pedrosa, Rachael C. Feord, Claudia Clopath, Ole Paulsen

Correspondence

arueda@mrc-lmb.cam.ac.uk (A.G.-R.),
op210@cam.ac.uk (O.P.)

In Brief

González-Rueda et al. show that presynaptic activation during slow-wave-sleep-like activity *in vivo* causes synaptic depression, unless it contributes to postsynaptic spiking. This plasticity rule offers an attractive mechanism for circuit refinement that improves signal-to-noise ratio and preserves previously stored information.

González-Rueda et al., 2018, Neuron *97*, 1244–1252
March 21, 2018 © 2018 The Author(s). Published by Elsevier Inc.
https://doi.org/10.1016/j.neuron.2018.01.047

Activity-Dependent Downscaling of Subthreshold Synaptic Inputs during Slow-Wave-Sleep-like Activity *In Vivo*

Ana González-Rueda,[1,2,*] Victor Pedrosa,[3,4] Rachael C. Feord,[1] Claudia Clopath,[3] and Ole Paulsen[1,5,*]
[1]Department of Physiology, Development and Neuroscience, University of Cambridge, Cambridge, CB2 3EG, UK
[2]Neurobiology Division, Medical Research Council (MRC) Laboratory of Molecular Biology, Cambridge, CB2 0QH, UK
[3]Department of Bioengineering, Imperial College London, London, SW7 2AZ, UK
[4]CAPES Foundation, Ministry of Education of Brazil, Brasilia, 70040-020, Brazil
[5]Lead Contact
*Correspondence: arueda@mrc-lmb.cam.ac.uk (A.G.-R.), op210@cam.ac.uk (O.P.)
https://doi.org/10.1016/j.neuron.2018.01.047

SUMMARY

Activity-dependent synaptic plasticity is critical for cortical circuit refinement. The synaptic homeostasis hypothesis suggests that synaptic connections are strengthened during wake and downscaled during sleep; however, it is not obvious how the same plasticity rules could explain both outcomes. Using whole-cell recordings and optogenetic stimulation of presynaptic input in urethane-anesthetized mice, which exhibit slow-wave-sleep (SWS)-like activity, we show that synaptic plasticity rules are gated by cortical dynamics *in vivo*. While Down states support conventional spike timing-dependent plasticity, Up states are biased toward depression such that presynaptic stimulation alone leads to synaptic depression, while connections contributing to postsynaptic spiking are protected against this synaptic weakening. We find that this novel activity-dependent and input-specific downscaling mechanism has two important computational advantages: (1) improved signal-to-noise ratio, and (2) preservation of previously stored information. Thus, these synaptic plasticity rules provide an attractive mechanism for SWS-related synaptic downscaling and circuit refinement.

INTRODUCTION

During development synaptic connections are formed, pruned, and refined following synaptic plasticity rules, the most prominent candidate of which is Hebbian plasticity. Hebb postulated that synapse modifications occur when presynaptic activity leads to, or correlates with, postsynaptic action potentials (spikes; Hebb, 1949). Hebbian forms of plasticity, including spike-timing-dependent plasticity (STDP), have since been extensively studied *in vitro* (Bi and Poo, 1998; Feldman, 2000) and incorporated in many computational models of circuit refinement during development (Song and Abbott, 2001; Clopath and Gerstner, 2010; Clopath et al., 2010; van Ooyen, 2011). However, to what extent these rules apply in the intact mammalian brain is not known.

The synaptic homeostasis hypothesis (Tononi and Cirelli, 2003) proposes that, whereas sensory experience at wake leads to strengthening of the associated neocortical synapses, slow-wave sleep (SWS) leads to a net depression of synaptic weights (Vyazovskiy et al., 2008; Liu et al., 2010). While Hebbian plasticity, such as STDP, could explain the sensory-dependent strengthening of synapses and underlie the emergence of neuronal assemblies during wake, it is not obvious how the same synaptic plasticity rules could also explain synaptic weakening during sleep. Indeed, it is not known whether the SWS-related downscaling of synaptic weights is due to synapse-specific mechanisms or more global, neuron-wide downscaling of synaptic weights (Turrigiano et al., 1998).

During SWS, cortical networks fluctuate at low frequency (<1 Hz) between periods of high activity, known as Up states, and more quiescent periods, known as Down states (Steriade et al., 1993). Up and Down states (UDS) are observed in single cells as subthreshold oscillations of up to 20 mV, leading to occasional firing exclusively during Up states. In contrast, during awake attention and rapid eye movement (REM) sleep, the cortex is characterized by asynchronous and irregular activity. Thus, it is possible that one role of UDS during SWS is to modulate synaptic plasticity rules in order to promote the appropriate downscaling of synapses during sleep.

Here we compared synaptic plasticity rules during Up and Down states in the developing barrel cortex of urethane-anesthetized mice showing SWS-like dynamics (Contreras and Steriade, 1997). We studied layer (L)4-L2/3 connections at postnatal days (P)16–P21, corresponding to the end of the critical period of development of this synapse, when maximal circuit refinement and sparsification of inputs are seen (Stern et al., 2001; Itami and Kimura, 2012; van der Bourg et al., 2017). We discovered that plasticity rules are modulated by Up states: spike-timing-dependent potentiation (t-LTP) is absent and active synapses failing to contribute to postsynaptic spiking are selectively depressed. We show in a computational model that this synaptic downscaling mechanism promotes the elimination of weak and

 © 2018 The Author(s). Published by Elsevier Inc.
This is an open access article under the CC BY license (http://creativecommons.org/licenses/by/4.0/).
Check for updates

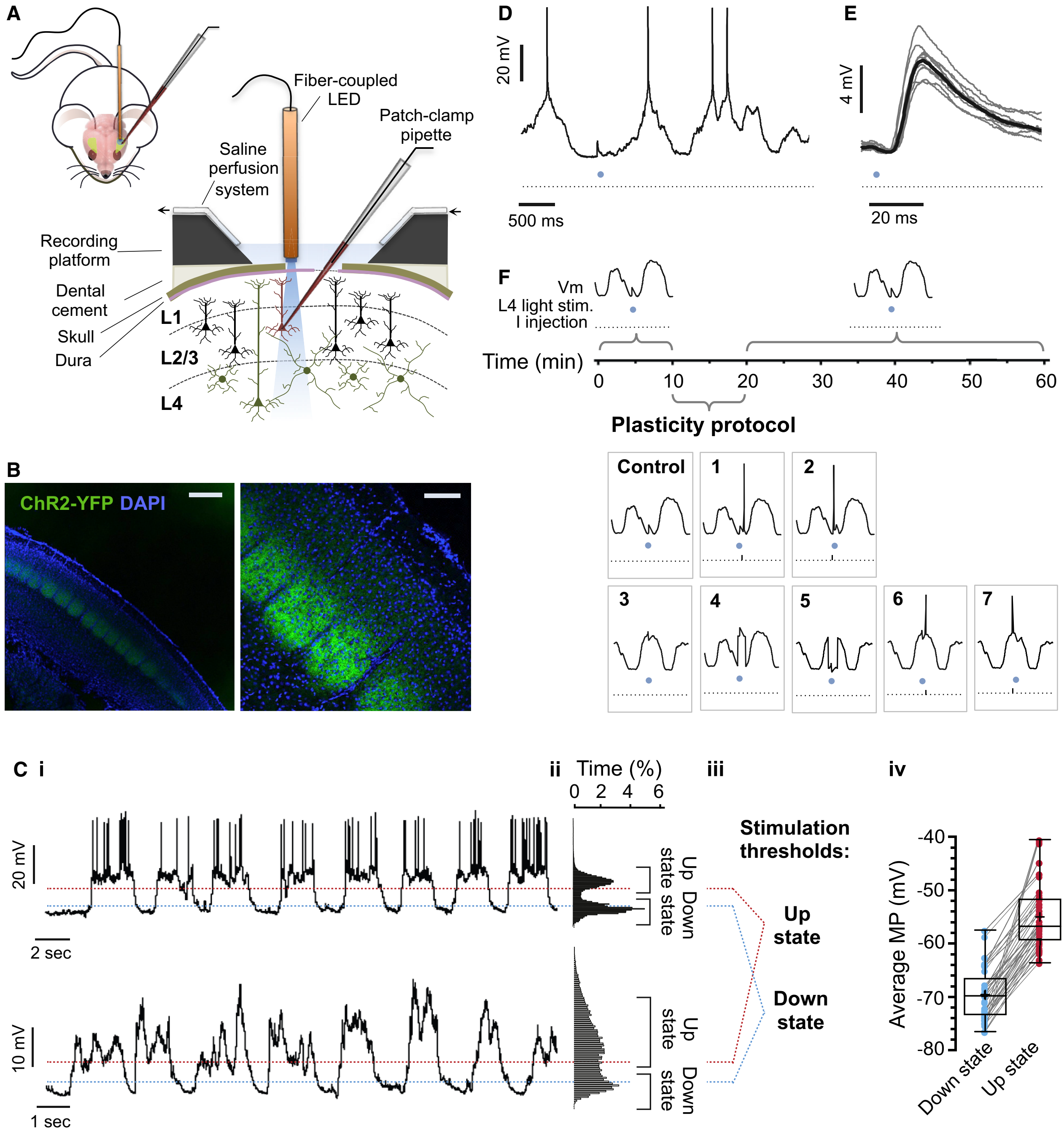

Figure 1. Monitoring L4 to L2/3 Synaptic Strength *In Vivo*

(A) Schematic of the technical approach. L2/3 neurons of urethane-anesthetized Six3-cre/Ai32 mice were recorded in whole-cell mode and ChR2-expressing L4 afferent fibers were activated using a fiber-coupled LED.

(B) Pattern of expression of ChR2 in the barrel cortex (scale bars: left, 500 μm; right, 200 μm).

(Ci–Civ) (Ci) Example trace of highly active L2/3 neuron (top) and sparsely spiking L2/3 neuron (bottom). (Cii) Bimodal distribution of membrane potential (MP). (Ciii) Thresholds for stimulation during Up states and Down states were 0.5 mV negative to the mean Up state MP (red dotted line) and 0.5 mV positive to the mean Down state MP (blue dotted line), respectively. (Civ) Mean MP at Up states and Down states for all cells recorded (n = 92 neurons in N = 81 mice). Box-and-whisker plots represent maximum, upper quartile, mean (cross), median, lower quartile, and minimum values.

(D) Example trace of light-evoked EPSP during Down state. A closed-loop was used to elicit EPSPs only at MP negative to Down state threshold. Spikes are truncated for clarity. See also Figures S1 and S2.

(E) Ten overlaid traces of light-evoked EPSPs during Down states (gray) and their mean (black).

(legend continued on next page)

preservation of strong synapses, thus enhancing signal-to-noise ratio (S/N).

RESULTS

Study of Synaptic Plasticity *In Vivo*

STDP-like synaptic changes *in vivo* have been previously described using sensory-evoked postsynaptic responses (Meliza and Dan, 2006; Jacob et al., 2007; Gambino and Holtmaat, 2012; Pawlak et al., 2013). While such studies are important to understand sensory coding, the specific inputs involved in each trial are unknown. To study synapse-specific plasticity *in vivo*, we used an LED for optogenetic activation of presynaptic afferents during whole-cell recording of L2/3 regular spiking neurons (Figures 1A and S1) in the barrel cortex of Six3-cre/Ai32 urethane-anesthetized mice expressing channelrhodopsin-2 (ChR2) selectively in L4 neurons (Figure 1B). The resting membrane potential of every L2/3 neuron recorded presented low-frequency (0.7 ± 0.06 Hz, n = 92) fluctuations between Up and Down states (−55 mV versus −69 mV, Figure 1C). Occasional REM-like activity was observed, characterized as long-lasting activated states (>5 s) (Figure S2). Firing of L2/3 neurons was sparse (0.25 Hz versus 1.06 Hz in L4, Figure S2) and restricted to activated states, with only few highly active neurons (7 out of 92; Figure 1C; de Kock and Sakmann, 2009). Excitatory postsynaptic potentials (EPSPs) were recorded in postsynaptic L2/3 neurons following light-stimulation of L4 fibers. To monitor EPSPs, we stimulated L4 at 0.1 Hz only during Down states using a closed-loop circuit to prevent stimulation during activated states (Figures 1C–1E). To assess synaptic plasticity, we applied a pairing protocol (100 repetitions at <0.2 Hz) following a 10-min stable baseline. At the end of the plasticity protocol, the Down state stimulation was resumed for a further 20 to 30 min (Figure 1F). Continued low-frequency light stimulation of L4 input during Down states over 50 min did not produce any significant change in synaptic weight (98% ± 6%, n = 13, one sample Student's t test p = 0.77, Figures 2A–2D), allowing us to use this method to study synaptic plasticity *in vivo*.

Conventional STDP during Down States

We first asked whether STDP can be induced during Down states *in vivo* by protocols similar to those described *ex vivo* in the absence of network activity (Feldman, 2000; Rodríguez-Moreno and Paulsen, 2008). When L4 light stimulation was followed within 10 ms by a single postsynaptic spike during Down states, t-LTP was induced (129% ± 15%, n = 8, versus 98% ± 6% interleaved controls, n = 13; two-sample Student's t test p = 0.038; Figures 2A–2D). Conversely, when light stimulation was preceded within 10 ms by a single postsynaptic spike, significant timing-dependent long-term depression (t-LTD) was induced (48% ± 11%, n = 5 versus 98% ± 6%, n = 13; two-sample Student's t test p = 0.0012; Figures 2A–2D). Thus, spike pairing during Down states induces conventional STDP *in vivo*, validating previous *ex vivo* results. However, neurons rarely spike during Down states (Figure 1C) implying that pairings of single pre- and postsynaptic spikes would not naturally occur during these periods. Thus, we next asked whether the same plasticity rules apply during Up states.

Synaptic Depression during Up States

In order to investigate how Up states influence synaptic plasticity, we first modified the closed-loop procedure to stimulate L4 afferents only during Up states in the plasticity protocol (100 repetitions at <0.2 Hz, protocol 3 in Figure 1F; fewer than 10% of stimulations occurred during activated states lasting >5 s, which might correspond to REM-like episodes, Figure S2). Surprisingly, we observed significant synaptic depression (63% ± 6%, n = 10, one-sample Student's t test p = 0.0002; Figure 3A), which was confirmed in Scnn1a-cre/Ai32 mice expressing ChR2 only in excitatory L4 neurons (51% ± 8%, n = 6, one-sample Student's t test p = 0.001; Figure S3).

Both experiments and computational models suggest that the membrane potential of the postsynaptic neuron can modify STDP induction (Sjöström et al., 2004; Clopath and Gerstner, 2010; Clopath et al., 2010). To test whether pairing of presynaptic spiking and subthreshold depolarization of L2/3 neurons was sufficient to induce synaptic plasticity, we paired presynaptic stimulation during Down state with a 500 ms positive current step, starting 250 ms before L4 stimulation (protocol 4 in Figure 1F), yielding a somatic depolarization equivalent to the mean potential during Up states for that neuron. This protocol did not induce plasticity (103% ± 13%, n = 6, one-sample Student's t test p = 0.83; Figure 3B). Conversely, pairing L4 stimulation during Up states with hyperpolarization to Down state level (protocol 5 in Figure 1F) did not prevent the induction of LTD (48% ± 8%, n = 6, one-sample Student's t test p = 0.0011; Figure 3B), suggesting that postsynaptic membrane voltage does not control Up state-associated synaptic depression.

Presynaptic *N*-methyl-D-aspartate receptor (NMDAR) activation is required for the induction of t-LTD at L4-L2/3 synapses of the barrel cortex (Bender et al., 2006; Rodríguez-Moreno and Paulsen, 2008). To investigate whether NMDARs are necessary also for *in vivo* Up state-associated LTD, we applied the NMDAR antagonist 2-amino-5-phosphonopentanoate (AP5; 0.2 mM) to the surface of the cortex. AP5 blocked depression (92% ± 5%, n = 7, one-sample Student's t test p = 0.17; Figure 3C). Equivalent results were obtained with extracellular application of the NMDAR channel blocker MK801 (30 μM) (91% ± 4%, n = 7, one-sample Student's t test p = 0.09; Figure 3C). However, MK801 loaded in the postsynaptic cell did not prevent Up state-mediated

(F) Diagram of the experimental design. EPSPs were monitored for 10 min using a 2-ms light pulse only during Down states (<0.1 Hz). Subsequently, one of eight protocols was applied (100 repetitions at <0.2 Hz): light stimulation at Down states only (control); light-stimulation at Down states followed (1) or preceded (2) by postsynaptic spike; light pulse during Up states only (3); light pulse during Down states paired with postsynaptic depolarization (4); light pulse during Up states paired with postsynaptic hyperpolarization (5); and presynaptic light stimulation during Up states followed (6) or preceded (7) by a postsynaptic spike. Following the plasticity protocol, EPSP was monitored by light stimulation during Down states (<0.1 Hz) for 20 to 30 min.

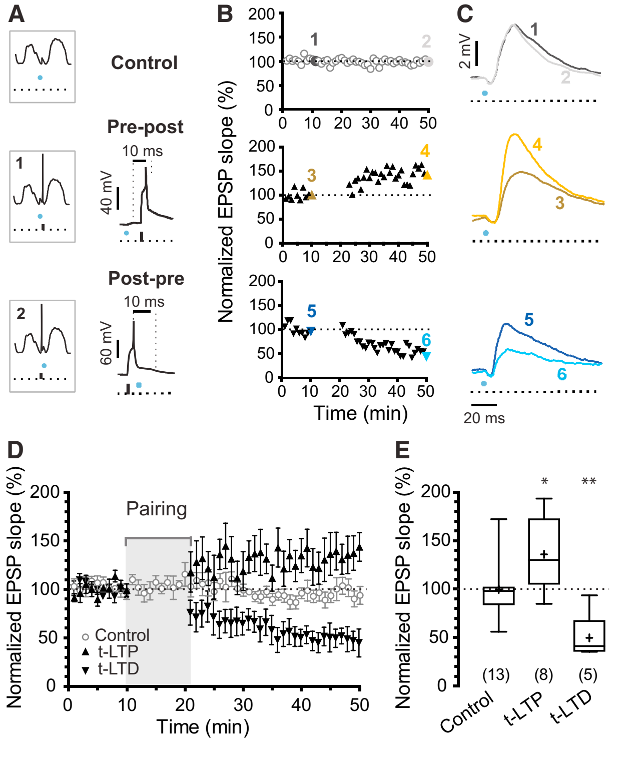

Figure 2. Synapse-Specific STDP Induced Using Light Stimulation during Down States *In Vivo*

(A) Diagram of the protocols applied: top, control; middle, protocol 1 (pre-post); bottom, protocol 2 (post-pre).

(B) Examples of each of the three experiments in (A). Symbols show 1-min means of the EPSP slopes normalized to the 10-min baseline.

(C) Representative mean traces from the 10th (1, 3, and 5) and 50th min (2, 4, and 6) of recording.

(D) Evoked EPSPs were stable over 50 min (<0.1 Hz, control; n = 13 cells in N = 10 mice, mean ± SEM). A pre-post pairing protocol applied during Down states led to t-LTP (n = 8 neurons in N = 8 mice, mean ± SEM), while a post-pre pairing protocol led to t-LTD (n = 5 neurons in N = 5 mice, mean ± SEM).

(E) Summary of the results in (D). Number of recordings indicated in parentheses. Mean EPSP slope from last 5 min of recording was normalized to mean EPSP slope of the last 5 min of the baseline. The box and whisker plots represent maximum, upper quartile, mean (cross), median, lower quartile, and minimum values. One-way ANOVA and Dunnett's post hoc test, $*p < 0.05$, $**p < 0.01$.

depression (44% ± 11%, n = 6, one-sample Student's t test p = 0.0033; Figure 3C). These results demonstrate that non-postsynaptic ionotropic NMDARs are required for Up state-associated LTD and that plasticity rules distinct from conventional STDP operate during Up states.

To test whether postsynaptic spikes following presynaptic activity during Up states (protocol 6 in Figure 1F) induces potentiation, as seen during Down states, we elicited a single spike in the postsynaptic L2/3 neuron 10 ms after presynaptic L4 light-stimulation during Up states. While no significant potentiation was observed, synapses were protected from depression (100% ± 9%, n = 7, one-sample Student's t test p = 0. 87; Figure 3E). This protection was not effective when a postsynaptic spike was evoked 50 ms following presynaptic stimulation (53% ± 8%, n = 5, one-sample Student's t test p = 0.0038; Figure 3E) or 10 ms prior to L4 stimulation during Up states (66% ± 7%, n = 7, one sample Student's t test p = 0.0041; Figure 3E), indicating a relatively narrow time window for protection against depression (Figure 3F).

Up State-Mediated Depression Could Explain Input-Specific Downscaling during SWS

The Up state-specific synaptic plasticity rule uncovered here would be consistent with the synaptic homeostasis hypothesis, which implies that synapses are selectively downscaled during SWS. However, in contrast to a global rescaling mechanism (Turrigiano et al., 1998), the rule uncovered here requires presynaptic activity during Up states. To test the implications of this plasticity rule, we developed a network model of 100 independently driven leaky integrate-and-fire presynaptic L4 neurons, projecting onto a single postsynaptic L2/3 neuron (Figure 4A). Synaptic weights were initially set at comparable values (0.2 ± 0.02, Figures 4B and 4C). To model synaptic weight changes during wake and successive sleep, we divided the simulation into two phases: "wake" and "sleep." To create a wake representation in L4-L2/3 connections ("sensory experience"), during the first half of the simulation, 5 of the L4 neurons received a 50% stronger external drive, and synaptic weights were updated according a conventional STDP rule (Figure 4B). This resulted in an overall potentiation of synaptic weights with stronger potentiation of connections from L4 neurons that received stronger input, due to the boosted coincidence of pre-postsynaptic pairings (Figure 4C), and in an increase in S/N (from 1.1 to 2.5, Figure 4D). To mimic synaptic weight changes during SWS in the subsequent sleep simulation, all L4 neurons received a comparable input drive and synaptic weights were updated with the Up state-specific synaptic plasticity rule uncovered here, such that L4 spikes alone led to depression unless followed within 10 ms by a L2/3 spike (Figure 4B). This SWS-like period resulted in the preservation of the highest synaptic weights, corresponding to the representation created during "wake," the depression of all other synaptic weights (Figure 4C), and a further increase in S/N (to 11.2, Figure 4D). The depression or maintenance of synaptic weights was independent of the synaptic plasticity rules during "wake" and only depended on the synaptic weights before "sleep" and the consequential probability of L4-L2/3 coincidence, which correlated to their firing rates (Figure S4).

To test the impact of the initial synaptic weight on the amount of synaptic weakening endured during SWS in our model, the initial synaptic weights were first set between 0.1 and 1 and the "sleep" simulation was run. Weak synapses were strongly depressed while stronger synapses remained unchanged (Figure 4E), consistent with theoretical predictions (Hashmi et al., 2013; Nere et al., 2013) and ultrastructural data (de Vivo et al., 2017).

Intuitively, the requirement of input activity for synaptic depression, combined with the protection against depression by postsynaptic spikes, would preserve previously stored synaptic representations despite presentation of new input patterns. Thus, we tested whether the new learning rule would retain previously stored input patterns while increasing the S/N for a new set

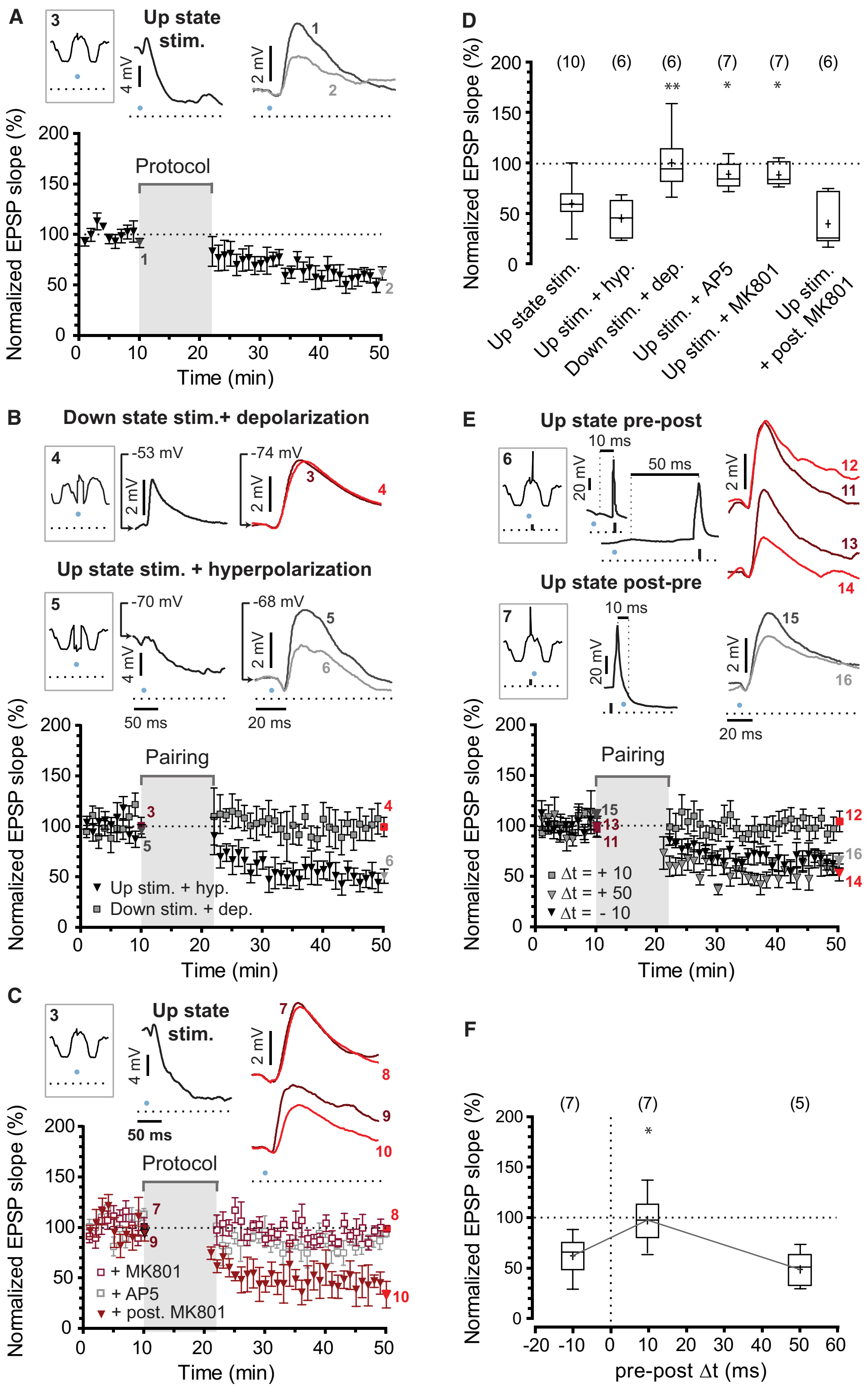

(legend on next page)

CellPress

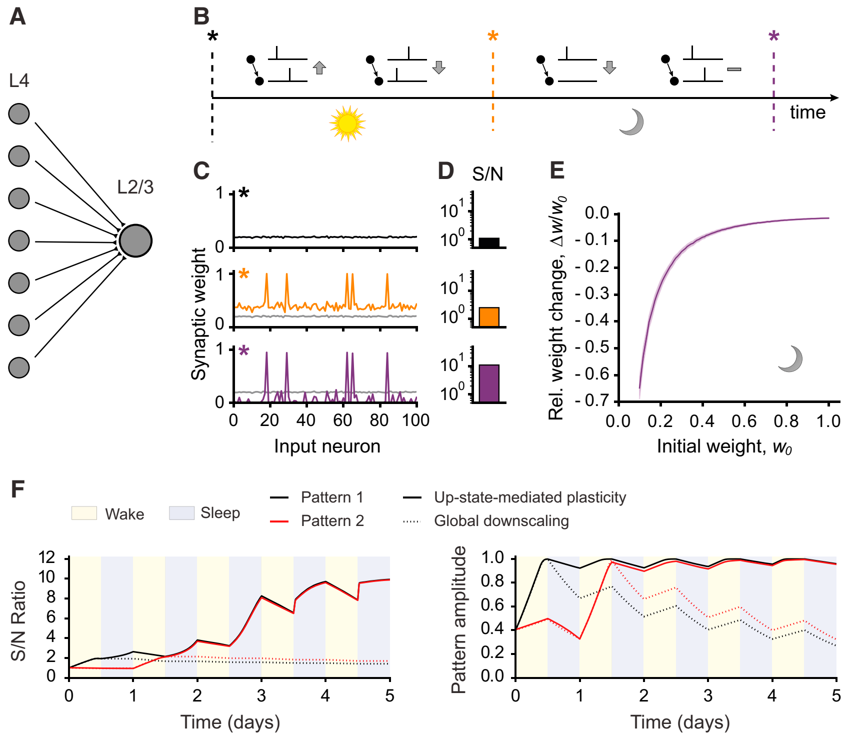

Figure 4. Up State-Mediated Depression Leads to Circuit Refinement in Model Network following Simulated Wake and Sleep

(A) Model of a feedforward network with 100 presynaptic L4 neurons projecting onto one single postsynaptic L2/3 neuron.

(B) Simulation protocol. Synaptic weights from all connections were initiated with comparable amplitude (black star). For the first half of the simulation ("wake," sun symbol, 800 s), synaptic weights were updated according to a conventional STDP rule. All presynaptic neurons received an external input to promote spiking and five of them received a 50% stronger input. During the second half ("sleep," moon symbol, 800 s), synaptic weights were updated with the Up state-mediated synaptic plasticity rule.

(C) Synaptic weights for three stages of the simulation. Top, black: start (black star in B). Middle, orange: after "wake" (orange star in B). Bottom, purple: end (purple star in B).

(D) Signal to noise ratio (S/N) for the three stages in (C).

(E) Relative weight change, $\Delta w/w_0$, plotted as a function of the initial synaptic weight, w_0, after Up state-mediated (sleep) plasticity. 200 simulations were averaged and the shaded area represents the SD.

(F) Simulated Up state-mediated plasticity preserves and enhances previously stored patterns. At day 0, five presynaptic neurons received 50% stronger input (pattern 1). At day 1, neurons from pattern 1 did not receive any extra external input, but another set of five neurons did (pattern 2). All presynaptic neurons received comparable external input and fired at the same rate from day 2. Left: evolution of S/N for both patterns (1, black; 2, red). Right: evolution of both pattern amplitudes. During "wake" (yellow), synaptic weights were updated according STDP. During "sleep" (purple), synaptic weights were updated according to either Up state-modulated plasticity (solid lines) or homogeneous synaptic scaling rule (dotted lines). Curves show a mean over 50 trials. See also Figure S4.

of inputs by repeating the "wake" and "sleep" phases over 5 days. A first set of inputs was strengthened at day 0 (pattern 1, Figure 4F). During the following "sleep" phase, all weights were updated either according the Up state-specific plasticity rule or an homogeneous global downscaling rule. As expected, the S/N increased and the amplitude of the pattern was conserved only following Up state-specific plasticity (Figure 4F). At day 1, a different set of inputs was strengthened (pattern 2). We observed that both patterns were preserved with increased S/N even after several subsequent wake/sleep cycles. In contrast, both S/N and pattern amplitude gradually decayed when a global scaling rule was used (Figure 4F). Thus, compared to global downscaling, this new plasticity rule promotes an increase in S/N and the retention of previously stored input patterns.

Figure 3. Up States Modulate the Induction of Synaptic Plasticity

(A) Presynaptic stimulation during Up states led to significant LTD (n = 10 cells in N = 10 mice, mean ± SEM). Schematic of the stimulation protocol (protocol 3, top left), representative traces of the plasticity protocol (black trace, top middle), and mean traces from the 10th (1) and 50th min (2) of example experiment are shown (see also Figure S3).

(B) Pairing of presynaptic stimulation during Down state and postsynaptic depolarization to Up state level (−53 mV in the example, protocol 4, top left and middle) failed to induce LTD (n = 6 neurons in N = 5 mice), while LTD was still induced when presynaptic stimulation during Up states was paired with postsynaptic hyperpolarization to Down state level (−70 mV in the example, protocol 5, n = 6 cells in N = 6 mice). Mean traces before (3 and 5) and after (4 and 6) plasticity protocol are shown.

(C) Up state-mediated LTD was prevented by AP5 (n = 7 cells in N = 7 mice) and MK801 (n = 7 neurons in N = 6 mice). Postsynaptic loading of MK801 failed to prevent LTD (n = 6 cells in N = 6 mice). Schematic of the stimulation protocol (protocol 3, top left), representative traces of the plasticity protocol (black trace, top middle), and the average traces from the 10th (7) and 50th min (8) of one of the experiments after MK801 application and with MK801 in the recording pipette (9 and 10, respectively) are shown.

(D) Summary of the results in (A), (B), and (C). Number of cells are indicated in parentheses. Mean EPSP slope from the last 5 min of recording was normalized to mean EPSP slope of the last 5 min of the baseline. Box-and-whisker plots represent maximum, upper quartile, mean (cross), median, lower quartile, and minimum values. One-way ANOVA and Dunnett's post hoc test, *p < 0.05, **p < 0.01.

(E) LTD was prevented by postsynaptic spikes following presynaptic stimulation within 10 ms (Up state pre-post pairing, Δt = +10, gray squares, n = 7 neurons in N = 7 mice), while LTD was still present if the pre-post time-window was widened to 50 ms (Up state pre-post pairing, Δt = +50, gray triangles, n = 5 cells in N = 5 mice) or reversed (Up state post-pre pairing, Δt = −10, black triangles, n = 7 neurons in N = 7 mice).

(F) Summary of results in (E) represented as in (D).

DISCUSSION

Our study shows that cortical network activity influences synaptic plasticity rules. Specifically, conventional STDP applies during slow-wave-sleep-like activity *in vivo* only during Down states. Synaptic stimulation during Up states invariably led to NMDAR-dependent synaptic depression unless the postsynaptic neuron spiked within a narrow time window following presynaptic stimulation, which protected against synaptic weakening. Incorporated into a computational network model, two important advantages of this new plasticity rule compared to conventional global rescaling were demonstrated: (1) improved S/N and (2) preservation of previously stored input patterns.

The induction of STDP during Down states is consistent with a huge body of experimental work in brain slices (e.g., Feldman, 2000; Rodríguez-Moreno and Paulsen, 2008). However, the physiological relevance of such STDP could be questioned, since cortical networks are relatively quiescent during Down states. Fewer and divergent studies have investigated synaptic plasticity following the pairing of presynaptic inputs and spontaneous or electrically evoked persistent activity in acute brain slices (Kruskal et al., 2013; Bartram et al., 2017), highlighting the importance of studying synaptic plasticity during activated states *in vivo*. During *in vivo* Up states, when spikes are more likely to occur, presynaptic-postsynaptic spike correlations were not required for synaptic plasticity, as subthreshold synaptic input was sufficient to induce NMDAR-dependent LTD. This may be a prominent form of plasticity *in vivo* owing to the low firing rate in L2/3 (Figure S2). LTD independent of postsynaptic spikes has previously been reported in slice preparations from neocortex, when presynaptic stimulation was paired with subthreshold postsynaptic depolarization (Sjöström et al., 2004), and even without any postsynaptic activity (Rodríguez-Moreno et al., 2013). Although our evidence suggests that postsynaptic depolarization is not required for Up state-associated LTD *in vivo*, we cannot exclude the possibility that local dendritic depolarization during Up states might be involved. Alternatively, the conditions for activation of presynaptic NMDARs might be met during Up state activity without the need for postsynaptic activity (Rodríguez-Moreno et al., 2013).

Our results highlight the importance of network activity in gating synaptic plasticity and indicate a bias toward synaptic depression during cortical slow oscillations. The plasticity rule uncovered here provides a mechanism by which connections that are strengthened during wake would be protected from depression, rather than further strengthened. This could be especially important during development as studied here, when neuronal connections are expected to depress (Feldman, 2000; Itami and Kimura, 2012; Banerjee et al., 2014). Interestingly, however, Up state-associated synaptic depression was also seen in adult mice (P30–P50, 71% ± 9%, n = 8 versus control 99% ± 9%, n = 6; Figure S3), suggesting that this form of plasticity could also contribute to circuit reshaping in mature cortex.

Network activity under urethane anesthesia resembles that during natural sleep (Contreras and Steriade, 1997; Clement et al., 2008). To what extent changes in neuromodulators are preserved during urethane anesthesia is not well studied; however, it has been reported that cholinergic modulation is comparable between urethane anesthesia sleep-like transitions and natural sleep (Steriade et al., 1993; Manns et al., 2000). Nevertheless, we cannot exclude the possibility that neuromodulatory systems are differentially regulated and it will be important to confirm our findings during natural sleep in future experiments. Although the vast majority of stimulations in the Up state plasticity protocols happened during SWS-associated Up states of short duration, a few stimulations occurred during REM-sleep-like episodes, and, although we find it unlikely, we cannot rule out that stimulations during REM-like episodes could have a disproportionate effect on synaptic weights. Still, our results offer a possible mechanism for the finding that sleep favors synaptic depression.

The Up state-associated plasticity studied here only takes account of pre- and postsynaptic spikes. Intuitively, if spiking activity was the only determinant of plasticity, the higher firing rate of L4 neurons compared to that of L2/3 neurons (Figure S2) would promote depression on its own. However, other mechanisms, such as subthreshold input cooperation within dendritic segments (Golding et al., 2002; Losonczy et al., 2008; Lee et al., 2016) could also promote the protection of synaptic connections even in the absence of postsynaptic spikes. Similarly, mechanisms other than those uncovered here could allow for the depression of highly efficacious synapses that would otherwise be permanently protected.

In our computational model, the Up state-associated synaptic plasticity rule effectively refined a previously acquired representation via the selective downscaling of synapses with lower synaptic weights, which is attractive as a mechanism for activity-dependent synaptic downscaling during sleep. Our data could explain how the occurrence of UDS during SWS induces selective downscaling of synaptic weights in an NMDAR-dependent manner without the need for any additional external input. The protection against depression by postsynaptic spikes suggests a mechanism by which spiking Hebbian assemblies maintain their synaptic weights (“neurons that fire together wire together”), while non-participating connections are depressed. This would also result in the sparsification of neuronal activity, which is seen during critical periods of development (van der Bourg et al., 2017). This mechanism also allows consolidation of new neuronal representations while conserving previously stored ones. In conclusion, we suggest that Up state-associated synaptic depression is a strong candidate for a mechanism to contribute to activity-dependent synaptic downscaling during SWS.

STAR★METHODS

Detailed methods are provided in the online version of this paper and include the following:

- KEY RESOURCES TABLE
- CONTACT FOR REAGENT AND RESOURCE SHARING
- EXPERIMENTAL MODEL AND SUBJECT DETAILS
- METHOD DETAILS
  - Surgery
  - Electrophysiology

- Thalamocortical slices
- Optogenetics and plasticity experiments
- Pharmacology
- Immunohistochemistry
- Analysis of synaptic plasticity
- Computational model
- Signal/noise analysis
- QUANTIFICATION AND STATISTICAL ANALYSIS
- DATA AND SOFTWARE AVAILABILITY

SUPPLEMENTAL INFORMATION

Supplemental Information includes four figures and can be found with this article online at https://doi.org/10.1016/j.neuron.2018.01.047.

ACKNOWLEDGMENTS

We thank Dr. G. Oliver for the Six3-cre#69 mouse line, Drs. M. Borel and J.L. Butler for sharing computer code, and Dr. A. Hay for technical and practical assistance. A.G.-R. held a Foster Studentship. R.C.F. is on the Cambridge BBSRC Doctoral Training Programme. V.P. is supported by CAPES Foundation, process n. 99999.001758/2015-02. C.C. holds a Wellcome Trust Investigator Award (200790/Z/16/Z). Research grants from the BBSRC to C.C. and O.P. are gratefully acknowledged.

AUTHOR CONTRIBUTIONS

A.G.-R. and O.P. conceived the experiments; A.G.-R. and R.C.F. performed and analyzed the electrophysiological recordings; V.P. and C.C. developed and implemented the computational model; and A.G.-R. and O.P. wrote the paper.

DECLARATION OF INTERESTS

The authors declare no competing interests.

Received: June 14, 2017
Revised: December 19, 2017
Accepted: January 26, 2018
Published: March 1, 2018

REFERENCES

Banerjee, A., González-Rueda, A., Sampaio-Baptista, C., Paulsen, O., and Rodríguez-Moreno, A. (2014). Distinct mechanisms of spike timing-dependent LTD at vertical and horizontal inputs onto L2/3 pyramidal neurons in mouse barrel cortex. Physiol. Rep. *2*, e00271.

Bartram, J., Kahn, M.C., Tuohy, S., Paulsen, O., Wilson, T., and Mann, E.O. (2017). Cortical Up states induce the selective weakening of subthreshold synaptic inputs. Nat. Commun. *8*, 665.

Bender, V.A., Bender, K.J., Brasier, D.J., and Feldman, D.E. (2006). Two coincidence detectors for spike timing-dependent plasticity in somatosensory cortex. J. Neurosci. *26*, 4166–4177.

Bi, G.Q., and Poo, M.M. (1998). Synaptic modifications in cultured hippocampal neurons: dependence on spike timing, synaptic strength, and postsynaptic cell type. J. Neurosci. *18*, 10464–10472.

Clement, E.A., Richard, A., Thwaites, M., Ailon, J., Peters, S., and Dickson, C.T. (2008). Cyclic and sleep-like spontaneous alternations of brain state under urethane anaesthesia. PLoS ONE *3*, e2004.

Clopath, C., and Gerstner, W. (2010). Voltage and spike timing interact in STDP – a unified model. Front. Synaptic Neurosci. *2*, 25.

Clopath, C., Büsing, L., Vasilaki, E., and Gerstner, W. (2010). Connectivity reflects coding: a model of voltage-based STDP with homeostasis. Nat. Neurosci. *13*, 344–352.

Contreras, D., and Steriade, M. (1997). State-dependent fluctuations of low-frequency rhythms in corticothalamic networks. Neuroscience *76*, 25–38.

Craig, M.T., Mayne, E.W., Bettler, B., Paulsen, O., and McBain, C.J. (2013). Distinct roles of GABAB1a- and GABAB1b-containing GABAB receptors in spontaneous and evoked termination of persistent cortical activity. J. Physiol. *591*, 835–843.

de Kock, C.P., and Sakmann, B. (2009). Spiking in primary somatosensory cortex during natural whisking in awake head-restrained rats is cell-type specific. Proc. Natl. Acad. Sci. USA *106*, 16446–16450.

de Vivo, L., Bellesi, M., Marshall, W., Bushong, E.A., Ellisman, M.H., Tononi, G., and Cirelli, C. (2017). Ultrastructural evidence for synaptic scaling across the wake/sleep cycle. Science *355*, 507–510.

Feldman, D.E. (2000). Timing-based LTP and LTD at vertical inputs to layer II/III pyramidal cells in rat barrel cortex. Neuron *27*, 45–56.

Furuta, Y., Lagutin, O., Hogan, B.L., and Oliver, G.C. (2000). Retina- and ventral forebrain-specific Cre recombinase activity in transgenic mice. Genesis *26*, 130–132.

Gambino, F., and Holtmaat, A. (2012). Spike-timing-dependent potentiation of sensory surround in the somatosensory cortex is facilitated by deprivation-mediated disinhibition. Neuron *75*, 490–502.

Golding, N.L., Staff, N.P., and Spruston, N. (2002). Dendritic spikes as a mechanism for cooperative long-term potentiation. Nature *418*, 326–331.

Hashmi, A., Nere, A., and Tononi, G. (2013). Sleep-dependent synaptic down-selection (II): single-neuron level benefits for matching, selectivity, and specificity. Front. Neurol. *4*, 148.

Hebb, D.O. (1949). The Organization of Behavior (Wiley).

Itami, C., and Kimura, F. (2012). Developmental switch in spike timing-dependent plasticity at layers 4-2/3 in the rodent barrel cortex. J. Neurosci. *32*, 15000–15011.

Jacob, V., Brasier, D.J., Erchova, I., Feldman, D., and Shulz, D.E. (2007). Spike timing-dependent synaptic depression in the in vivo barrel cortex of the rat. J. Neurosci. *27*, 1271–1284.

Kruskal, P.B., Li, L., and MacLean, J.N. (2013). Circuit reactivation dynamically regulates synaptic plasticity in neocortex. Nat. Commun. *4*, 2574.

Lee, K.F., Soares, C., Thivierge, J.P., and Béïque, J.C. (2016). Correlated synaptic inputs drive dendritic calcium amplification and cooperative plasticity during clustered synapse development. Neuron *89*, 784–799.

Liu, Z.-W., Faraguna, U., Cirelli, C., Tononi, G., and Gao, X.B. (2010). Direct evidence for wake-related increases and sleep-related decreases in synaptic strength in rodent cortex. J. Neurosci. *30*, 8671–8675.

Losonczy, A., Makara, J.K., and Magee, J.C. (2008). Compartmentalized dendritic plasticity and input feature storage in neurons. Nature *452*, 436–441.

Manns, I.D., Alonso, A., and Jones, B.E. (2000). Discharge properties of juxtacellularly labeled and immunohistochemically identified cholinergic basal forebrain neurons recorded in association with the electroencephalogram in anesthetized rats. J. Neurosci. *20*, 1505–1518.

Meliza, C.D., and Dan, Y. (2006). Receptive-field modification in rat visual cortex induced by paired visual stimulation and single-cell spiking. Neuron *49*, 183–189.

Nere, A., Hashmi, A., Cirelli, C., and Tononi, G. (2013). Sleep-dependent synaptic down-selection (I): modeling the benefits of sleep on memory consolidation and integration. Front. Neurol. *4*, 143.

Pawlak, V., Greenberg, D.S., Sprekeler, H., Gerstner, W., and Kerr, J.N.D. (2013). Changing the responses of cortical neurons from sub- to suprathreshold using single spikes in vivo. eLife *2*, e00012.

Rema, V., Armstrong-James, M., and Ebner, F.F. (1998). Experience-dependent plasticity of adult rat S1 cortex requires local NMDA receptor activation. J. Neurosci. *18*, 10196–10206.

Rodríguez-Moreno, A., and Paulsen, O. (2008). Spike timing-dependent long-term depression requires presynaptic NMDA receptors. Nat. Neurosci. *11*, 744–745.

Rodríguez-Moreno, A., González-Rueda, A., Banerjee, A., Upton, A.L., Craig, M.T., and Paulsen, O. (2013). Presynaptic self-depression at developing neocortical synapses. Neuron *77*, 35–42.

Sjöström, P.J., Turrigiano, G.G., and Nelson, S.B. (2004). Endocannabinoid-dependent neocortical layer-5 LTD in the absence of postsynaptic spiking. J. Neurophysiol. *92*, 3338–3343.

Song, S., and Abbott, L.F. (2001). Cortical development and remapping through spike timing-dependent plasticity. Neuron *32*, 339–350.

Steriade, M., Nuñez, A., and Amzica, F. (1993). Intracellular analysis of relations between the slow (< 1 Hz) neocortical oscillation and other sleep rhythms of the electroencephalogram. J. Neurosci. *13*, 3266–3283.

Stern, E.A., Maravall, M., and Svoboda, K. (2001). Rapid development and plasticity of layer 2/3 maps in rat barrel cortex in vivo. Neuron *31*, 305–315.

Tononi, G., and Cirelli, C. (2003). Sleep and synaptic homeostasis: a hypothesis. Brain Res. Bull. *62*, 143–150.

Turrigiano, G.G., Leslie, K.R., Desai, N.S., Rutherford, L.C., and Nelson, S.B. (1998). Activity-dependent scaling of quantal amplitude in neocortical neurons. Nature *391*, 892–896.

van der Bourg, A., Yang, J.W., Reyes-Puerta, V., Laurenczy, B., Wieckhorst, M., Stüttgen, M.C., Luhmann, H.J., and Helmchen, F. (2017). Layer-specific refinement of sensory coding in developing mouse barrel cortex. Cereb. Cortex *27*, 4835–4850.

van Ooyen, A. (2011). Using theoretical models to analyse neural development. Nat. Rev. Neurosci. *12*, 311–326.

Vyazovskiy, V.V., Cirelli, C., Pfister-Genskow, M., Faraguna, U., and Tononi, G. (2008). Molecular and electrophysiological evidence for net synaptic potentiation in wake and depression in sleep. Nat. Neurosci. *11*, 200–208.

STAR★METHODS

KEY RESOURCES TABLE

REAGENT or RESOURCE	SOURCE	IDENTIFIER
Chemicals, Peptides, and Recombinant Proteins		
AP5	Tocris Bioscience	Cat.# 0106
MK801	Tocris Bioscience	Cat.# 0924
DNQX	Tocris Bioscience	Cat.# 0189
Biocytin	Sigma-Aldrich	Cat.# B4261
Streptavidin, Alexa 633 Fluor-conjugated	Thermo Fisher Scientific, Molecular Probes	Cat.# S21375
Experimental Models: Organisms/Strains		
Mouse: B6;129S-Gt(ROSA)26Sortm32(CAG–COP4*H134R/eYFP)Hze/J mice (Ai32)	The Jackson Laboratory	JAX: 012569; RRID: IMSR_JAX:012569
Mouse: Tg(Six3-Cre)69Frty/GcoJ (Six3-cre)	Furuta et al., 2000; (also available at the Jackson Laboratory)	N/A; RRID: IMSR_JAX:019755
Mouse: B6;C3-Tg(Scnn1a-cre)3Aibs/J	The Jackson Laboratory	JAX: 00961; RRID: IMSR_JAX:009613
Software and Algorithms		
Igor Pro	Wavemetrics	https://www.wavemetrics.com/products/igorpro/igorpro.htm
Python	N/A	The code for all the simulations will be submitted to ModelDB after publication

CONTACT FOR REAGENT AND RESOURCE SHARING

Further information and any requests for resources should be directed to and will be fulfilled by Lead Contact Prof. Ole Paulsen (op210@cam.ac.uk).

EXPERIMENTAL MODEL AND SUBJECT DETAILS

Tg(Six3-Cre)69Frty/GcoJ (Six3-cre) mice were crossed with B6;129S-Gt(ROSA)26Sortm32(CAG–COP4*H134R/eYFP)Hze/J mice (Ai32; Jackson Laboratory, Maine, USA) in order to express ChR2(H134R)-YFP in L4 neurons of the somatosensory cortex (Furuta et al., 2000). For a subset of experiments Ai32 mice were crossed with B6;C3-Tg(Scnn1a-cre)3Aibs/J (Scnn1a-Cre; Jackson Laboratory, Maine, USA). Unless otherwise stated, only Six3-cre/Ai32 mice ranging from P15 to P21 were used for experiments. Both males and females were used for experiments. Animals were housed on a 12-hr light/dark cycle and the mother was fed *ad libitum*. The research was performed under the Animals (Scientific Procedures) Act 1986 Amendment Regulations 2012 following ethical review by the University of Cambridge Animal Welfare and Ethical Review Body (AWERB).

METHOD DETAILS

Surgery

Mice were anesthetized with an intraperitoneal injection of urethane (1 g per kg of body weight). Upon cessation of reflexes, the top of the head of the animal was shaved and the skin covering the right hemisphere was removed. A blue LED light was used to identify and mark over the skull the YFP-fluorescent barrel field. A head-restraining platform was cemented (Super-Bond C & B; Prestige Dental, Bradford, UK) to the skull around the mark indicating the barrel cortex location. The animal was placed on the recording frame over a low-noise heating pad (FHC, Termobit Prod. srl., Bucharest, Romania) to aid body temperature maintenance. A 200-500 μm craniotomy was performed at the marked spot and the dura mater was carefully removed from a small area of the craniotomy (20-100 μm). Care was taken to avoid bleeding or drying of the meninges and brain tissue. Saline (0.9% NaCl) was superfused constantly with a 2 mL/minute laminar flow using a peristaltic pump.

Electrophysiology

Whole-cell current-clamp recordings were obtained from L2/3 neurons of the primary somatosensory cortex of urethane-anesthetized mice using 6-9 MΩ pipettes pulled from borosilicate glass capillaries (1B120F-4, World Precision Instruments, Stevenage, UK). Pipettes were filled with artificial intracellular solution containing (in mM): K-gluconate 150, HEPES 10, NaCl 4, ATP-Mg 4, GTP-Na 0.3 and EGTA 0.2; adjusted to pH 7.2 and osmolarity 270-290 mOsm/L. Data were recorded using an Axon Multiclamp 700B amplifier (Molecular Devices, Union City, CA, USA). Signals were low-pass filtered at 2 kH and acquired at 5 kHz using a data interface ITC-18 AD board (Instrutech, Port Washington, NY, USA) on a PC running Igor Pro (Wavemetrics, Lake Oswego, OR, USA). A reference silver pellet electrode (A-M Systems, Carlsborg, WA, USA) was placed in the saline bath covering the craniotomy. The recording pipette was controlled using a micromanipulator at a 50° inclination. High positive pressure (>500 mbar) was applied and the pipette was lowered to the surface of the brain. A 5-ms-long square pulse of voltage of 4 to 8 mV at 100 Hz was delivered via the recording electrode. The pipette was quickly advanced 100 μm to L2 of the barrel cortex. The pressure was lowered to 60 mbar and the pipette was advanced in 2 μm steps. Cell contact produced a small reduction (around 10%) in resistance of the pipette, which could be seen as a proportional decrease in the size of the step of current in the oscilloscope. Cell contact also produced a pulsation artifact or flickering in the positive values of the current recorded. If no cell contact was detected after 250 steps of 2 μm, the pipette was retracted and a new pipette was used to record a new cell. Recordings were discarded if the access resistance was >25 MΩ or changed >10% along the recording. For a subset of experiments, single unit recordings of L2/3 and L4 neurons were done using 3-4 MΩ tungsten electrodes (Microelectrodes, Cambridge, UK) paired with 1 MΩ tungsten electrodes for L2/3 LFP recording.

Thalamocortical slices

Thalamocortical slices of 350 μm thickness containing the barrel subfield of somatosensory cortex were prepared as previously described (Rodríguez-Moreno et al., 2013; Banerjee et al., 2014) Briefly, mice were decapitated under isoflurane anesthesia and the brain was rapidly removed and placed in ice-cold artificial cerebrospinal fluid (aCSF) with the following composition (in mM): NaCl 126, KCl 3, NaH_2PO_4 1.25, $MgSO_4$ 2, $CaCl_2$ 2, $NaHCO_3$ 26, glucose 10, pH 7.2-7.4; bubbled with carbogen gas (95% O_2/5% CO_2) and with an osmolarity adjusted to 280-300 mOsm/L. The brain was placed on a 10° ramp rostral side down and a vertical cut was made through the tissue at an angle of 55° to the anteroposterior axis of the brain. Slices from the right somatosensory cortex were cut using a vibratome (VT 1200S, Leica, Wetzlar, Germany) and maintained in a submerged-style chamber at room temperature until used. The flow rate of aCSF in the recording chamber was 2 mL/min.

Optogenetics and plasticity experiments

L4 neurons were excited using a 470 nm fiber-coupled LED system (150 μm, Thorlabs, Ely, UK). The LED fiber was positioned centrally above the recorded cell on top of the dura mater. Light intensity was adjusted to produce 3-6 mV EPSPs at Down states. The average membrane potential during Down states and Up states was measured over 10 s prior to the start of the plasticity protocol. The average membrane potential at Down states plus 0.5 mV was used as the Down state threshold, whereas the average membrane potential at Up states minus 0.5 mV was used as threshold for Up states. In order to trigger a light-pulse only at Down states and at <0.1 Hz, the membrane potential was scanned in 10-ms time bins using a closed-loop. When the membrane potential crossed the threshold for Down states a 10 s recording was triggered and a 2-ms light-pulse was delivered 10 ms into the recording. At the end of the 10 s-recording the closed-loop scanning was resumed in order to detect the next Down state. Each light stimulus was time stamped in order to obtain minute-averages of the EPSP slope. Following a 10-minute baseline recording, a plasticity protocol was applied at <0.2 Hz for 100 repeats. Following the plasticity protocol, the stimulus during Down states were resumed in order to assess changes in the EPSP slope.

For the experiments performed in acute brain slices, a monopolar stainless steel stimulation electrode was used to elicit EPSPs in L2/3 as control. Electrical stimulation was delivered with 2 s delay from the light-pulse.

Pharmacology

In a subset of experiments, NMDA receptors were blocked by epidural administration of 0.2 mM AP5 or 30 μM MK801 (Tocris Bioscience, Bristol, UK) via the superfusate, as previously described (Rema et al., 1998). In order to determine the reversal potential of the inhibitory component of ChR2 stimulation, 0.2 mM AP5 and 20 μM DNQX (Tocris Bioscience, Bristol, UK) were added to the superfusate.

Immunohistochemistry

Images from the barrel cortex of Six3-Cre/Ai32 mice and Scnn1a-Cre/Ai32 were taken to assess the level and location of ChR2 expression. Moreover, post hoc reconstruction of a subset of the neurons recorded *in vivo* was possible after biocytin (Sigma-Aldrich, Dorset, UK) staining. Biocytin was diluted at 5 mg/mL in the internal solution used for whole-cell recordings. At the end of the recordings, the brain was cut in 200 μm thalamocortical slices as previously described. Slices containing the barrel cortex were washed once in PBS and were left overnight in fixing solution containing 4% (w/v) PFA in PBS at 4°C. Slices were incubated in Alexa 633 Fluor-conjugated streptavidin (1:1000; Molecular Probes, Eugene, OR) in PBS and 0.3% Triton X-100 (Sigma-Aldrich, Dorset, UK) overnight at 4°C. Before mounting, slices were incubated for 2 min in DAPI in PBS. Images were taken with a confocal microscope (SP8, Leica, Wetzlar, Germany) and a 25X and a 40X objective and color intensity was adjusted using ImageJ.

Analysis of synaptic plasticity

To assess plasticity, EPSP slopes were used as a measure of synaptic strength. Slope measurements were made on the rising phase of the EPSP as a linear fit between time points corresponding to 25%–30% and 70%–75% of peak amplitude of the EPSP, or of the first slope in the case of polysynaptic EPSPs. EPSP slopes were averaged over 1-minute time bins. The change in EPSP slope after the plasticity protocol was expressed relative to EPSP slopes during the baseline recording. EPSP slopes were averaged over the last 5 min of the initial baseline and the last 5 min of recording, generally corresponding to min 25-30 after the plasticity induction protocol. For offline UDS analysis, the membrane potential noise level was used as previously described (Craig et al., 2013).

Computational model

We simulated a feedforward network composed of 100 presynaptic neurons projecting onto one postsynaptic neuron. We used the leaky integrate-and-fire neuron model. In this model, the membrane potential of a neuron is described by

$$\tau_m \frac{du}{dt} = -(u - u_{rest}) + RI(t),$$

where u_{rest} denotes the membrane voltage at rest, R denotes the membrane resistance, $I(t)$ denotes the external current and τ_m denotes the membrane time constant. If the membrane potential reaches a threshold u_{th} at time $t^{(f)}$, the membrane potential is reset to u_{reset} and we call $t^{(f)}$ the firing time. After being reset, the membrane potential follows the same equation again.

The term $I(t)$ takes into account all of the current being injected into a neuron; these can be from an external source or from other neurons. When a neuron fires it propagates a current to all other connected neurons. In order to model this current, we assumed that the conductance between a presynaptic neuron j and a postsynaptic neuron i increases instantaneously every time the presynaptic neuron fires, and decays exponentially otherwise:

$$\begin{aligned} g_j &\rightarrow g_j + 1 \quad && \text{if j fires and} \\ \frac{dg_j}{dt} &= -g_j/\tau_{syn} \quad && \text{otherwise,} \end{aligned}$$

where τ_{syn} is the synaptic time constant. The synaptic current was then calculated through

$$I^{syn}(t) = -w_{ij} g_j (u - E^{syn}),$$

where w_{ij} is the synaptic weight from neuron j to neuron i and E^{syn} is the synaptic reversal potential.

Each presynaptic neuron received an independent external current whose amplitude varied in time and was determined by a filtered Gaussian noise, with filtering time constant 20 ms. The postsynaptic neuron also received a constant external current.

For the wake phases of the simulations, the synaptic weights were updated by the conventional STDP rule, in which pre-post events lead to potentiation and post-pre events lead to depression. In order to implement this rule, we defined a presynaptic trace $\overline{x}_j$ (for each presynaptic neuron j) and a postsynaptic trace $\overline{y}$ that was incremented by 1 for each pre or postsynaptic spike, respectively, and decayed otherwise:

$$\begin{aligned} \overline{x}_j &\rightarrow \overline{x}_j + 1 \quad && \text{if presynaptic neuron j fires and} \\ \tau_+ \frac{d\overline{x}_j}{dt} &= -\overline{x}_j \quad && \text{otherwise,} \end{aligned}$$

and

$$\begin{aligned} \overline{y} &\rightarrow \overline{y} + 1 \quad && \text{if the postsynaptic neuron fires and} \\ \tau_- \frac{d\overline{y}}{dt} &= -\overline{y}_i \quad && \text{otherwise,} \end{aligned}$$

where τ_- is the depression time constant and τ_+ the potentiation time constant.

The synaptic weight w_j was then updated by the following:

$$\begin{aligned} w_j(t) &\rightarrow w_j(t) - A_- \overline{y}(t) \quad && \text{if } t = t^{pre}, \\ w_j(t) &\rightarrow w_j(t) + A_+ \overline{x}_j(t) \quad && \text{if } t = t^{post}. \end{aligned}$$

where A_- is the depression amplitude and A_+ is the potentiation amplitude. Synaptic weights were also bounded between 0 and 1: $0 < w < 1$.

During the sleep phases of our simulations, the synaptic weights were updated following either an Up state-mediated plasticity or an homogeneous synaptic scaling. For the Up state-mediated plasticity, synaptic weights were updated such that presynaptic spikes alone led to depression whereas presynaptic spikes followed within 10 ms by a postsynaptic action potential led to no change. In order to implement this rule, we defined a presynaptic trace $\overline{x}_j$ (for each presynaptic neuron j) that was reset to 10 for every presynaptic spike and decayed linearly otherwise obeying

$$\bar{x}_j \rightarrow 10 \qquad \text{if presynaptic neuron j fires and}$$
$$\tau \frac{d\bar{x}_j}{dt} = -1 \qquad \text{otherwise,}$$

where $\tau = 1$ ms. The synaptic weight w_j was then updated by the following:

$$w_j(t) \rightarrow w_j(t) - A \qquad \text{if } t = t^{pre},$$
$$w_j(t) \rightarrow w_j(t) + AH(\bar{x}_j(t)) \qquad \text{if } t = t^{post}.$$

where A is the depression amplitude and $H(x)$ is the Heaviside step function defined by $H(x) = 1$ if $x > 0$ and $H(x) = 0$ if $x < 0$. Therefore, a presynaptic spike leads to depression with amplitude A and a postsynaptic spike within 10 ms of the presynaptic spike protects against this depression (increases the synaptic weight be the same amount A). Synaptic weights were also bounded between 0 and 1: $0 < w < 1$.

The homogeneous synaptic scaling was implemented by downscaling all synaptic weights by the same amount. The weights were downscaled such that they would be reduced by 33% at the end of each sleep phase.

Signal/noise analysis

The S/N was measured as the mean amplitude of the synaptic weights from the neurons relative to a specific pattern divided by the average of all synaptic weights.

Parameter summary for simulations in Figure 4:

Name	Value	Description
Neuron model		
τ_m	10 ms	Membrane time constant
u_{th}	10 mV	Spiking threshold
u_{rest}	0 mV	Resting potential
E^{syn}	30 mV	Synaptic reversal potential
u_{reset}	0 mV	Value at which the potential is reset after a spike
T_{ref}	3 ms	Refractory time
Network and Synapse Model		
N_E	100	Size of presynaptic population
τ_E	10 ms	Decay constant of excitatory conductance
$\bar{g}_E$	1 nS	Peak synaptic conductance
Plasticity Model		
τ_{STDP}	20 ms	Decay constant of pre- and postsynaptic traces
A_+ (C-D)	1×10^{-3}	Amplitude of learning rate for pre-post events (conventional STDP) - Figures 4C and 4D
A_- (C-D)	1×10^{-3}	Amplitude of learning rate for post-pre events (conventional STDP) - Figures 4C and 4D
A (C-D and F)	1×10^{-3}	Amplitude of learning rate for presynaptic events (sleep plasticity) - Figures 4C, 4D, and 4F
A (e)	2×10^{-4}	Amplitude of learning rate for presynaptic events (sleep plasticity) - Figure 4E
A_+ (F)	2×10^{-5}	Amplitude of learning rate for pre-post events (conventional STDP) - Figure 4F
A_- (F)	2×10^{-5}	Amplitude of learning rate for post-pre events (conventional STDP) - Figure 4F
A (S4 C-D)	5×10^{-3}	Amplitude of learning rate for presynaptic events (sleep plasticity) - Figures S4C and S4D

QUANTIFICATION AND STATISTICAL ANALYSIS

The number of experimental recordings (“n”) and the number of animals (“N”) are indicated in the figure legends. When the “n” number per experimental condition was too low to assess the type of distribution, the data were assumed normal and a two-tailed one sample Student’s t test was used to assess long-term plasticity. In order to compare the magnitude of plasticity to a control condition

a two-tailed two sample Student's t test was used. For multiple comparisons to a single group, a one-way ANOVA followed by a Dunnett's test was performed.

DATA AND SOFTWARE AVAILABILITY

A Python code with our simulations will be submitted to ModelDB. We developed this code to simulate a feedforward network of excitatory neurons undergoing Up state-mediated plasticity. In our implementation of Up state-mediated plasticity, presynaptic spikes alone led to synaptic depression whereas pre- followed by postsynaptic spikes within 10 ms led to no change in synaptic weight. The simulations were used to confirm our intuition that the Up state-mediated plasticity leads to the refinement and protection of previously stored memories in a simulated neuronal network.

Neuron, Volume *97*

Supplemental Information

Activity-Dependent Downscaling of Subthreshold Synaptic Inputs during Slow-Wave-Sleep-like Activity *In Vivo*

Ana González-Rueda, Victor Pedrosa, Rachael C. Feord, Claudia Clopath, and Ole Paulsen

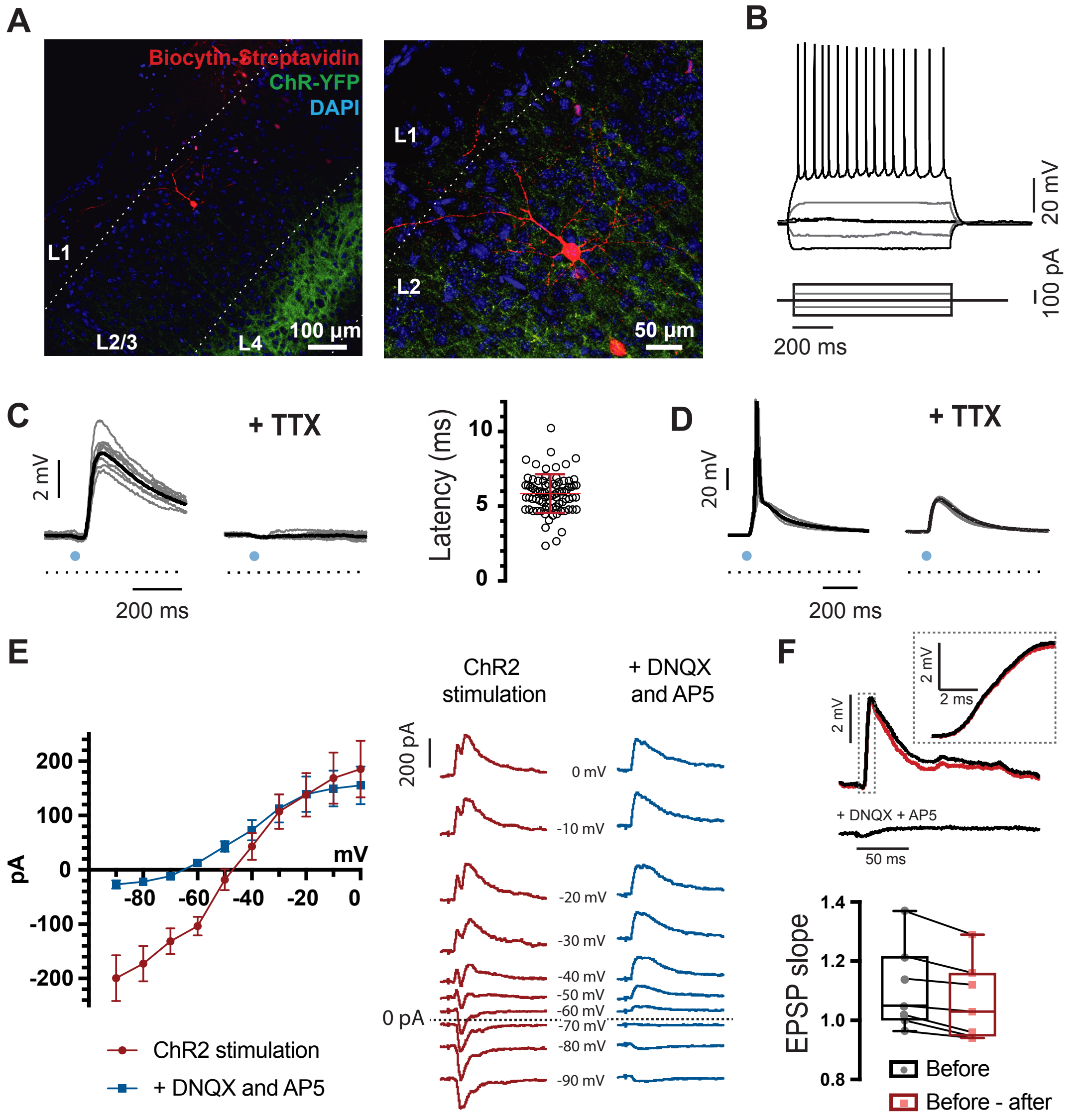

Figure S1 (related to Figure 1): Whole-cell patch clamp of L2/3 neurons in Six3-cre/Ai32 mice.

(**A**) Example L2/3 neuron recorded *in vivo* and filled with biocytin.

(**B**) Example response of the membrane potential of a regular spiking neuron recorded *in vivo* (top) to different steps of current (bottom).

(**C**) Light-evoked EPSPs recorded in L2/3 are completely eliminated when tetrodotoxin (TTX) is added to the superfusate. The average EPSP latency of all recordings is shown (n = 92, mean ± SD).

(**D**) Subthreshold depolarization by direct activation of ChR2 in L4 neurons is unaffected by TTX.

(**E**) Light-evoked EPSC current-voltage curve before (red) and after (blue) DNQX and AP5 application in acute brain slices. Light intensity was previously adjusted to produce a 4 mV EPSP at resting potential in current clamp. The reversal potential of the ChR2-mediated EPSC could be extrapolated at -47 mV and the inhibitory component at -66 mV. n = 7 cells in N = 3 mice.

(**F**) Example light-evoked EPSP before (black top) and after (black bottom)

DNQX and AP5 application in acute brain slices. The subtracted trace (before minus after) is shown in red. n = 7 cells in N = 3 mice. The box-and-whisker plots represent the maximum, upper quartile, mean (cross), median, lower quartile and minimum values and the values for individual neurons are overlaid. The GABA-mediated component of the EPSP could only account for 4% of the measured slope.

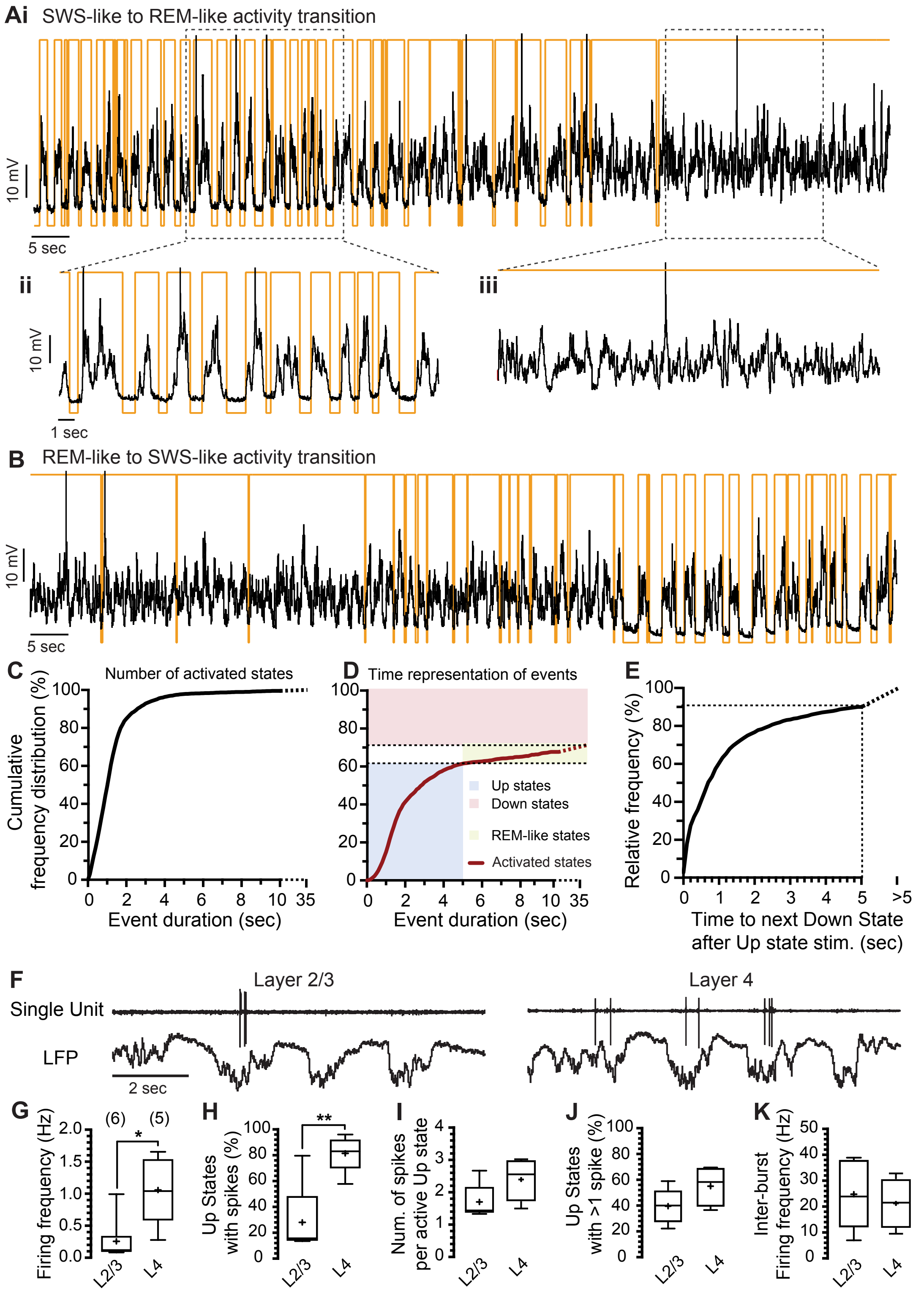

Figure S2 (related to Figure 1): Spontaneous activity in P16-P21 Six3-Cre/Ai32 mice under urethane anesthesia.

(**A**) Example of slow-wave-sleep (SWS)-like to rapid eye movement (REM)-like activity transition (**i**). The Up and Down state transitions are overlaid in orange and a close up of a SWS-like period (**ii**) and REM-like period (**iii**) are shown. Spikes were cut for display.

(**B**) Example of REM-like to SWS-like transition.

(**C**) Cumulative distribution of the number of activated states (Up states and

REM-like states) of different durations. The majority of activated events (84.5%) correspond to Up states lasting <2 seconds and only 2.2% of the events corresponded to putative REM-like states lasting >5 seconds. Data obtained from 30 minute recordings in n = 5 neurons in N = 4 mice.

(**D**) Time representation of Up states, putative REM-like states (lasting >5 seconds) and Down states (as analysed for **C**). Neurons spent 10.1% of the total time in REM-like states.

(**E**) Time to next Down state following Up state stimulation during Up state stimulation protocols (protocols 3, 5, 6 and 7). Only 9% of stimulations were done during putative REM-like states.

(**F**) Example traces of layer (L)2/3 and L4 single unit recordings paired with LFP recordings in L2/3. n = 6 L2/3 and n = 5 L4 neurons recorded in N = 4 mice.

(**G**) Firing frequency (in Hz) of L2/3 and L4 neurons.

(**H**) Percentage of Up states in which L2/3 and L4 were active.

(**I**) Number of spikes per Up state in which L2/3 or L4 were active (respectively).

(**J**) Percentage of Up states in which neurons spiked and produced more than one action potential.

(**K**) Inter-burst frequency when neurons were activated more than once in an Up state.

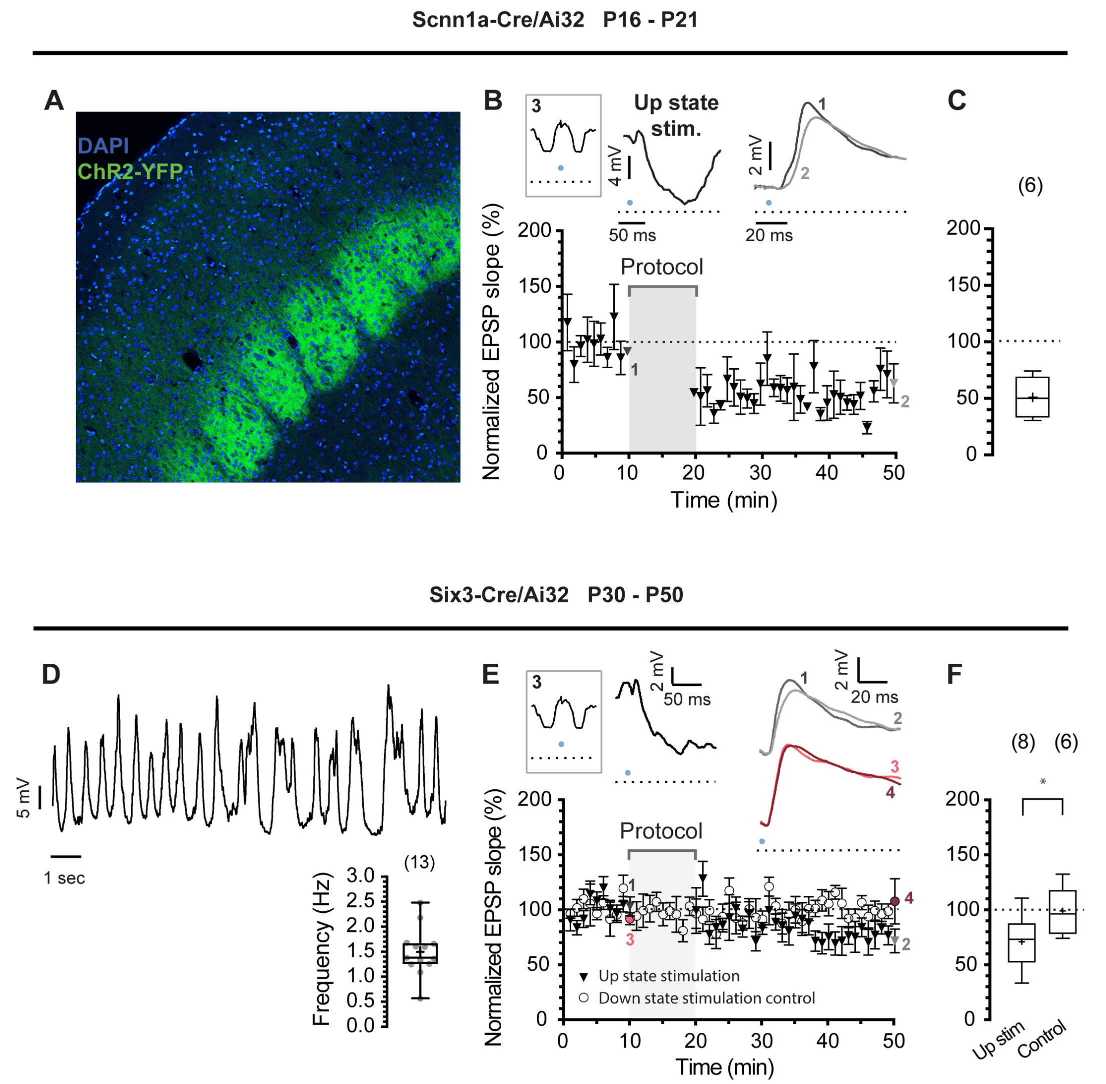

Figure S3 (related to Figure 3): Up state stimulation leads to L4 to L2/3 depression in a different mouse line and at a different age.

(**A**) ChR2 expression pattern in Scnn1a-cre/Ai32 mice.

(**B**) L4 light-stimulation during Up states leads to LTD in Scnn1a-cre/Ai32 mice. Schematic of the stimulation protocol (protocol 3, gray rectangle, top left), representative traces of the plasticity protocol (black trace, top middle) and the average traces from the 10th and 50th minute of an example experiment (1 and 2 respectively) are shown.

(**C**) Summary of result in B. The box-and-whisker plots represent the maximum, upper quartile, mean (cross), median, lower quartile and minimum values (n = 6 cells in N = 6 mice).

(**D**) Example of spontaneous Up and Down state (UDS) activity in P30-P50 Six3-cre/Ai32 mice under urethane anaesthesia. The UDS frequency for all neurons recorded at this age is shown (n = 13 neurons in N = 10 mice).

(**E**) L4 light-stimulation during Up states leads to depression (black triangles, n = 8 neurons in N = 8 mice) while no changes in synaptic weight are observed when presynaptic stimulation is performed during Down states (control, circles, n = 6 neurons in N = 5 mice).

(**F**) Summary of results in E. Two-tailed Student's t test. * $p < 0.05$.

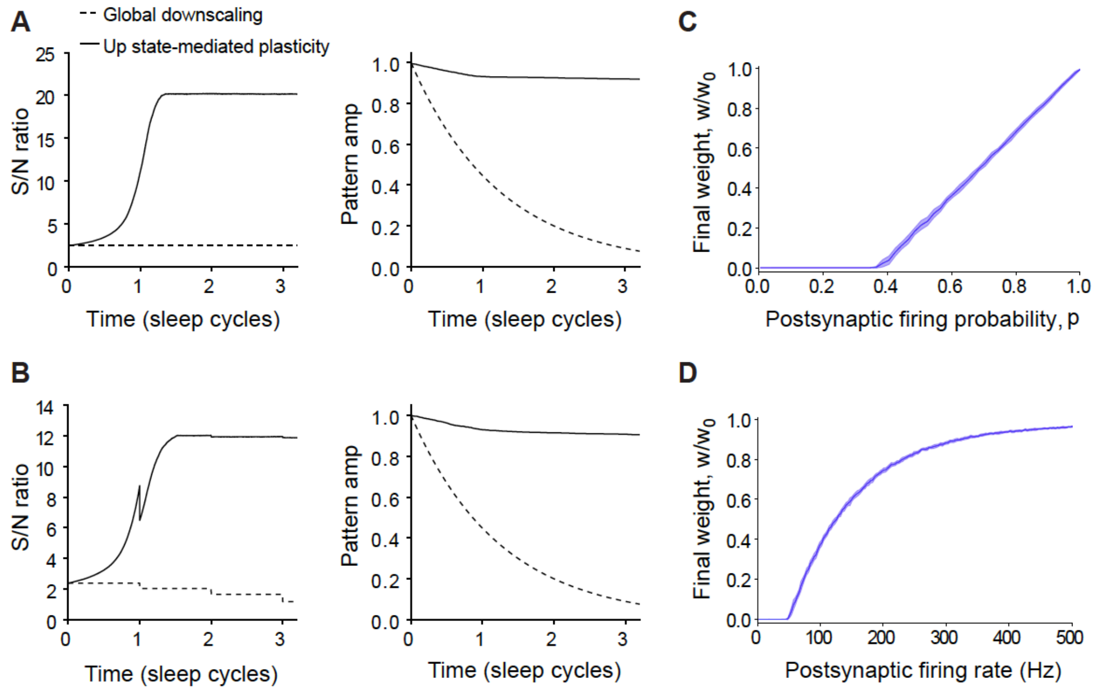

Figure S4 (related to Figure 4): Circuit refinement is independent of specific awake learning rule and synaptic depression can be prevented by postsynaptic activity.

(**A**-**B**) Simulation of a feedforward network composed of 100 presynaptic neurons (representing L4 neurons) projecting onto one single postsynaptic neuron (representing a L2/3 neuron), analogous to simulations in Figure 4. One 'wake' phase of learning was simulated. During this phase, 5 presynaptic neurons received 50% stronger inputs. The synaptic weights at the end of this simulation were used as the initial weight for the sleep phase. These simulations are analogous to the simulations performed in Figure 4F, but with different awake learning rules.

(**A**) Simulated Up state-mediated plasticity preserves and enhances previously stored patterns without learning between sleep cycles. During sleep cycles, all presynaptic neurons received comparable external input and fired at the same rate, on average. Left: evolution of S/N ratio over three sleep cycles. Right: evolution of pattern amplitudes. During sleep cycles, synaptic weights were updated following either the Up state-modulated plasticity (solid lines) or a homogeneous synaptic scaling rule (dashed lines). Between sleep cycles, synaptic weights were kept fixed. Curves show an average over 50 trials.

(**B**) Simulated Up state-mediated plasticity preserves and enhances previously stored patterns when a second pattern is strengthened between sleep cycles. The simulation was performed in the same way as in A but a second pattern was strengthened between sleep cycles. During sleep cycles, all presynaptic neurons received comparable external input and fired at the same rate, on average. Left: evolution of S/N ratio over three sleep cycles. Right: evolution of pattern amplitudes. During sleep cycles, synaptic weights

were updated following either the Up state-modulated plasticity (solid lines) or a homogeneous synaptic scaling rule (dashed lines). Between sleep cycles, the synaptic weights of a second set of presynaptic neurons (pattern 2) were reset to their maximum values. Curves show an average over 50 trials. Our simulations indicate that the Up state-mediated depression leads to circuit refinement independent of the specific rule underlying awake learning.

(**C**-**D**) We simulated a very simple feedforward network, with only one pre- and one postsynaptic neuron. The presynaptic neuron fired at 10 Hz and we assumed that the connection between the neurons was weak enough such that, at this rate, presynaptic spikes would not elicit postsynaptic action potentials.

(**C**) Ratio between final synaptic weight and initial synaptic weight as a function of p, where p is the probability of the postsynaptic neuron to fire within 10 ms after a presynaptic spike (and then prevent synaptic depression). The synapse was updated following the Up state-mediated plasticity. Curves show an average over 50 trials. Shaded areas represent one standard deviation from average.

(**D**) Ratio between final synaptic weight and initial synaptic weight as a function of the postsynaptic firing rate. Both neurons fired independently following a Poisson process. The synapse was updated following the Up state-mediated plasticity. Curves show an average over 50 trials. Shaded areas represent one standard deviation from average.
